# Learning predictive statistics from temporal sequences: Dynamics and strategies

**DOI:** 10.1167/17.12.1

**Published:** 2017-10-02

**Authors:** Rui Wang, Yuan Shen, Peter Tino, Andrew E. Welchman, Zoe Kourtzi

**Affiliations:** wangr@psych.ac.cn; Yuan.Shen@xjtlu.edu.cn; P.Tino@cs.bham.ac.uk; aew69@cam.ac.uk; zk240@cam.ac.uk; Key Laboratory of Mental Health, Institute of Psychology, Chinese Academy of Sciences, Beijing, China,; Department of Psychology, University of Cambridge, Cambridge, UK; Department of Mathematical Sciences, Xi'an Jiaotong-Liverpool University, Suzhou, China; School of Computer Science, University of Birmingham, Birmingham, UK; School of Computer Science, University of Birmingham, Birmingham, UK; Department of Psychology, University of Cambridge, Cambridge, UK; Department of Psychology, University of Cambridge, Cambridge, UK

**Keywords:** *learning*, *behavior*, *vision*

## Abstract

Human behavior is guided by our expectations about the future. Often, we make predictions by monitoring how event sequences unfold, even though such sequences may appear incomprehensible. Event structures in the natural environment typically vary in complexity, from simple repetition to complex probabilistic combinations. How do we learn these structures? Here we investigate the dynamics of structure learning by tracking human responses to temporal sequences that change in structure unbeknownst to the participants. Participants were asked to predict the upcoming item following a probabilistic sequence of symbols. Using a Markov process, we created a family of sequences, from simple frequency statistics (e.g., some symbols are more probable than others) to context-based statistics (e.g., symbol probability is contingent on preceding symbols). We demonstrate the dynamics with which individuals adapt to changes in the environment's statistics—that is, they extract the behaviorally relevant structures to make predictions about upcoming events. Further, we show that this structure learning relates to individual decision strategy; faster learning of complex structures relates to selection of the most probable outcome in a given context (maximizing) rather than matching of the exact sequence statistics. Our findings provide evidence for alternate routes to learning of behaviorally relevant statistics that facilitate our ability to predict future events in variable environments.

## Introduction

Extracting structure from initially incomprehensible streams of events is fundamental to a range of human abilities, from navigating in a new environment to learning a language. These skills rely on identifying spatial and temporal regularities, often with minimal explicit feedback (Aslin & Newport, 2012; Perruchet & Pacton, 2006). The human brain appears expert at learning contingencies between co-occurring stimuli on the basis of mere exposure. For instance, structured patterns become familiar after simple exposure to items (shapes, tones, or syllables) that co-occur spatially or follow in a temporal sequence (Chun, 2000; Fiser & Aslin, 2002a; Saffran, Aslin, & Newport, 1996; Saffran, Johnson, Aslin, & Newport, 1999; Turk-Browne, Junge, & Scholl, 2005).

Previous work on human statistical learning has focused on repetitive patterns and associative pairings. However, event structures in the natural environment typically comprise regularities of variable complexity, from simple repetition to complex probabilistic combinations. For instance, when learning a new piece of music, we can make use of the intrinsic structure that ranges from tones to melodies (Fitch & Martins, 2014). Or, when reading about a new topic, we first extract information about the key components and then their interdependencies that together explain particular phenomena. To account for the full range of learning behaviors, we therefore need to understand the processes involved in extracting information of variable complexity.

**Figure 1:**
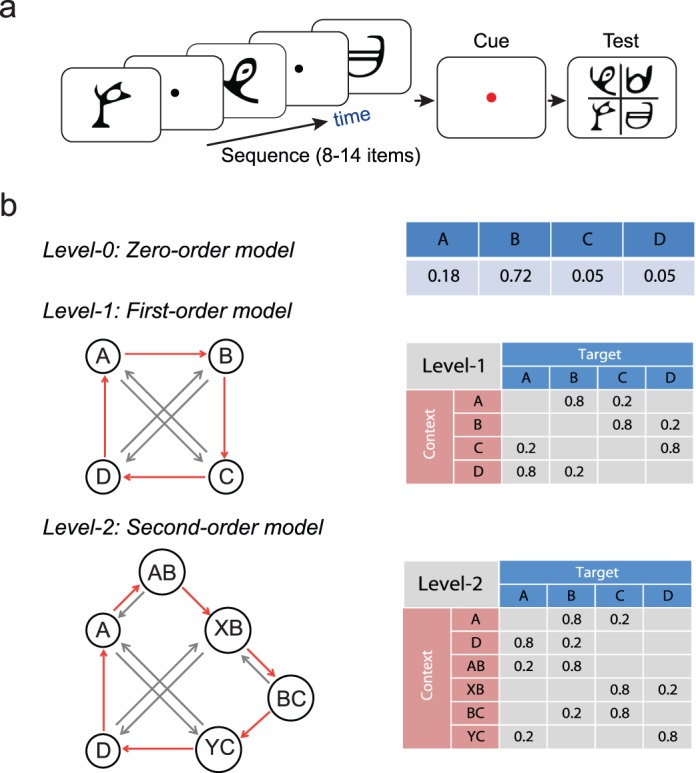
Trial and sequence design. (a) Eight to 14 symbols were presented one at a time in a continuous stream followed by a cue and the test display. (b) Sequence design. For the zero-order model (Level 0): Different states (A, B, C, D) are assigned to four symbols with different probabilities. For first- (Level 1) and second- (Level 2) order models, diagrams indicate states (circles) and conditional probabilities (red arrow: high; gray arrow: low). Transitional probabilities were arranged in a 4 × 4 (Level 1) or 4 × 6 (Level 2) conditional-probability matrix.

Here we investigate the dynamics of learning predictive structures by modeling whether and when participants extract the structure that governs temporal sequences that change in their complexity. To allow them to do so unencumbered by past experience, we tested participants with sequences of unfamiliar symbols, where the sequence structure changed unbeknownst to the participants (Figure 1). We increased sequence complexity by manipulating the memory order (i.e., context length) of the Markov model used to generate the sequences. In particular, we presented participants with sequences that were determined first by frequency statistics (i.e., occurrence probability per symbol) and then by more complex context-based statistics (i.e., the probability of a given symbol appearing depends on the preceding symbols). Participants performed a prediction task in which they indicated which symbol they expected to appear following exposure to a sequence of variable length. Following previous statistical learning paradigms, participants were exposed to the sequences without trial-by-trial feedback.

Our results show that the participants' ability to extract behaviorally relevant temporal statistics improved with training. To understand the dynamics of this structure learning, we track human predictions as they evolve over time (i.e., during exposure to the sequences). In particular, we compare the sequence of button presses made by the participants to the presented temporal sequences and test whether and when participants' judgments approximate the Markov model that produced the presented sequences. Using this approach, we show that participants adapt to changes in the environment's statistics and exploit previous knowledge of similar, but simpler, statistics when learning higher order structures. Further, we show that learning of predictive structures relates to individual decision strategy; some individuals used a probability-maximization strategy (i.e., extracting the most probable outcome in a given context), while others chose to match the exact sequence statistics. We demonstrate that faster learning of complex structures is associated with selecting the most probable outcomes in a given context, suggesting that attempting to learn all possible statistical contingencies may limit the ability to learn higher order structures.

## Material and methods

### Observers

We tested 50 participants (mean age = 22.9 years)—Experiment 1, Group 0: *n* = 19; Experiment 2, Group 1: *n* = 8; Group 2: *n* = 12; no-training control experiment, *n* = 11. All observers were unaware of the aim of the study, had normal or corrected-to-normal vision, and gave written informed consent. This study was approved by the University of Birmingham Ethics Committee.

### Stimuli

Stimuli comprised four symbols chosen from the Ndjuká syllabary (Figure 1a; Turk-Browne, Scholl, Chun, & Johnson, 2009). The symbols (black on a mid-gray background; size = 8.5°) were highly discriminable and were unfamiliar to the participants. Experiments were controlled using MATLAB and the Psychophysics Toolbox 3 (Brainard, 1997; Pelli, 1997). Stimuli were presented on a gamma-corrected 21-in. ViewSonic P225f monitor (1,280 × 1,024 pixel; 0.3 × 0.3 mm/pixel; 85-Hz refresh rate). Experiments were conducted in a dark room and the viewing distance was 45 cm.

### Sequence design

To generate probabilistic sequences that differed in complexity, we used a temporal Markov model and manipulated the memory order of the sequence, which we refer to as the context length. The Markov model determines a sequence of symbols, where the symbol at time *i* is determined probabilistically by the previous *k* symbols. We refer to the symbol *s*(*i*) presented at time *i* as the *target* and to the preceding *k*-tuple of symbols (*s*(*i* − 1), *s*(*i* − 2), …, *s*(*i* − *k*)) as the *context*. The value of *k* is the order or level of the model

\newcommand{\bialpha}{\boldsymbol{\alpha}}\newcommand{\bibeta}{\boldsymbol{\beta}}\newcommand{\bigamma}{\boldsymbol{\gamma}}\newcommand{\bidelta}{\boldsymbol{\delta}}\newcommand{\bivarepsilon}{\boldsymbol{\varepsilon}}\newcommand{\bizeta}{\boldsymbol{\zeta}}\newcommand{\bieta}{\boldsymbol{\eta}}\newcommand{\bitheta}{\boldsymbol{\theta}}\newcommand{\biiota}{\boldsymbol{\iota}}\newcommand{\bikappa}{\boldsymbol{\kappa}}\newcommand{\bilambda}{\boldsymbol{\lambda}}\newcommand{\bimu}{\boldsymbol{\mu}}\newcommand{\binu}{\boldsymbol{\nu}}\newcommand{\bixi}{\boldsymbol{\xi}}\newcommand{\biomicron}{\boldsymbol{\micron}}\newcommand{\bipi}{\boldsymbol{\pi}}\newcommand{\birho}{\boldsymbol{\rho}}\newcommand{\bisigma}{\boldsymbol{\sigma}}\newcommand{\bitau}{\boldsymbol{\tau}}\newcommand{\biupsilon}{\boldsymbol{\upsilon}}\newcommand{\biphi}{\boldsymbol{\phi}}\newcommand{\bichi}{\boldsymbol{\chi}}\newcommand{\bipsi}{\boldsymbol{\psi}}\newcommand{\biomega}{\boldsymbol{\omega}}\[P\left( {s\left( i \right)|s\left( {i - {\rm{1}}} \right),s\left( {i - {\rm{2}}} \right), \ldots ,s\left( {\rm{1}} \right)} \right){\rm{\ }} = P\left( {s\left( i \right)|s\left( {i - {\rm{1}}} \right),s\left( {i - {\rm{2}}} \right), \ldots ,s\left( {i - k} \right)} \right),k \lt i.\]

The simplest \newcommand{\bialpha}{\boldsymbol{\alpha}}\newcommand{\bibeta}{\boldsymbol{\beta}}\newcommand{\bigamma}{\boldsymbol{\gamma}}\newcommand{\bidelta}{\boldsymbol{\delta}}\newcommand{\bivarepsilon}{\boldsymbol{\varepsilon}}\newcommand{\bizeta}{\boldsymbol{\zeta}}\newcommand{\bieta}{\boldsymbol{\eta}}\newcommand{\bitheta}{\boldsymbol{\theta}}\newcommand{\biiota}{\boldsymbol{\iota}}\newcommand{\bikappa}{\boldsymbol{\kappa}}\newcommand{\bilambda}{\boldsymbol{\lambda}}\newcommand{\bimu}{\boldsymbol{\mu}}\newcommand{\binu}{\boldsymbol{\nu}}\newcommand{\bixi}{\boldsymbol{\xi}}\newcommand{\biomicron}{\boldsymbol{\micron}}\newcommand{\bipi}{\boldsymbol{\pi}}\newcommand{\birho}{\boldsymbol{\rho}}\newcommand{\bisigma}{\boldsymbol{\sigma}}\newcommand{\bitau}{\boldsymbol{\tau}}\newcommand{\biupsilon}{\boldsymbol{\upsilon}}\newcommand{\biphi}{\boldsymbol{\phi}}\newcommand{\bichi}{\boldsymbol{\chi}}\newcommand{\bipsi}{\boldsymbol{\psi}}\newcommand{\biomega}{\boldsymbol{\omega}}\(k = {0^{{\rm{th}}}}\) order model is a random memoryless source. This generates, at each time point *i*, a symbol according to symbol probability *P*(*s*), without taking account of the previously generated symbols.

The order *k* = 1 Markov model generates symbol *s*(*i*) at each time *i* conditional on the previous symbol *s*(*i* − 1). This introduces a memory in the sequence; that is, the probability of a particular symbol at time *i* depends on the preceding symbol *s*(*i* − 1). Unconditional symbol probabilities *P*(*s*(*i*)) for the case *k* = 0 are replaced with conditional ones *P*(*s*(*i*)|*s*(*i* − 1)).

We applied the same logic to higher memory orders: When *k* = 2, the probability of a symbol at time *i* depends on the two preceding symbols *s*(*i* − 1), *s*(*i* − 2): *P*(*s*(*i*)|*s*(*i* − 1), *s*(*i* − 2)). That is, the memory in the sequence is deeper and the number of conditioning contexts increases with memory depth *k*.

At each time point in the sequence, the symbol that follows a given context is determined probabilistically, making the Markov sequences stochastic. The underlying Markov model can be represented through the associated context-conditional target probabilities. We used four symbols that we refer to as stimuli A, B, C and D. The correspondence between stimuli and symbols was counterbalanced across participants.

For Level 0, the Markov model was based on the probability of symbol occurrence: One symbol had a high probability of occurrence and one low, while the remaining two symbols appeared rarely (Figure 1b). For example, the probabilities of occurrence for the four symbols A, B, C, and D were 0.18, 0.72, 0.05, and 0.05, respectively. Presentation of a given symbol was independent of the stimuli that preceded it.

For Level 1, the target depended on the immediately preceding stimulus (Figure 1b). Given a context (the last-seen symbol), only one of two targets could follow: One had a high probability of being presented and the other a low probability (e.g., 80% vs. 20%). For example, when symbol A was presented, only symbols B or C were allowed to follow, and B had a higher probability of occurrence than C.

For Level 2, the Markov model contained temporal contexts of variable length (Figure 1b), extending the Level 1 model. That is, the Markov model included both first- and second-order contexts (i.e., the target symbols depended on the preceding two symbols). As with the Level 1 model, given a specific context, only two symbols were allowed to follow, one with a high and one with a low probability (e.g., 80% vs. 20%). The target probabilities for contexts with B as the last symbol (i.e., AB, BB, CB, DB) were constrained by allowing only two sets of conditional target probabilities, namely *P*(*s*|AB) and *P*(*s*|XB), where *s* is the target symbol (A, B, C, or D) and X stands for any other symbol apart from A (i.e., B, C, or D). The same structure was imposed for second-order contexts with C as the last symbol. In this case, the two sets of conditional target probabilities were *P*(*s*|BC) and *P*(*s*|YB), where Y stands for any other symbol apart from B (i.e., A, C, or D). To discriminate between contexts that shared the same last symbol (i.e., XB vs. AB, and YC vs. BC), different targets were assigned to each context (one with high and one with low probability). For example, the allowed targets following XB were C and D, while the targets for context AB were B and A. To ensure that learning was not biased by differences in context probability, the four Level 1 contexts (A, B, C, D) appeared at an equal 25% probability, and the six Level 2 contexts (A, AB, XB, BC, YC, D) appeared at similar probabilities (0.19, 0.19, 0.16, 0.16, 0.15, and 0.15, respectively).

To test whether participants adapt to changes in the temporal structure, we ensured that the sequences across levels were matched for properties (i.e., number or identity of symbols) other than context length. Further, we designed the stochastic sources from which the sequences were generated so that the context-conditional uncertainty remained highly similar across levels. In particular, for the zero-order source only two symbols were likely to occur most of the time; the remaining two symbols had very low probability (0.05). This was introduced to ensure that there was no difference in the number of symbols presented across levels. Of the two dominant symbols, one was more probable (probability 0.72) than the other (probability 0.18). This structure is preserved in the Markov chain of order 1 or 2, where conditional on the previous symbols, only two symbols were allowed to follow, one with higher probability (0.80) than the other (0.20). This ensures that the structure of the generated sequences across levels differed predominantly in memory order (i.e., context length) rather than context-conditional probability.

### Experimental design

We generated probabilistic sequences of symbols that differed in their complexity using temporal Markov models—that is, sequences determined by simple frequency statistics (Level 0) and more complex sequences defined by context-based statistics (Levels 1 and 2). Manipulating the memory depth of the stochastic source that generated the sequences allowed us to systematically vary the context length of the sequences (Figure 1, Supplementary Material). In Experiment 1 (Group 0), we trained participants with sequences that changed in their complexity starting from Level 0 and then moving to Level 1 and Level 2 sequences. In Experiment 2, we tested two additional groups of participants: Group 1 trained first at Level 1 and then Level 2; Group 2 trained only at Level 2. For each level, observers completed a minimum of three and a maximum of five training sessions (840–1,400 trials). Training at each level ended when participants reached plateau performance (i.e., performance did not change significantly for two sessions). A posttraining test session followed training per level, during which observers were presented with sequences determined by the statistics of the trained level (90 trials). Before and after training (pre- and posttraining sessions), participants were tested with sequences from all three levels (30 trials per level). Overall, Group 0 completed 13–15 training sessions and five test sessions (on average 23.3 days); Group 1 completed 8–10 training sessions and four test sessions (on average 15.6 days); Group 2 completed four or five training sessions and three test sessions (on average 9.5 days). Further, to ensure that any changes observed across time were a result of active training, we performed a no-training control experiment. Specifically, participants were tested on all three levels in two behavioral sessions that were separated by a period (27.9 days on average) comparable to that between the pre- and posttraining sessions for Group 0. The stimuli, sequences, and procedure matched the first and last test sessions in Experiment 1, but no training took place between these two sessions.

#### Training sessions

Each training session comprised five blocks of structured sequences (56 trials per block) and lasted 1 hr. To ensure that sequences in each block were representative of the Markov-model order per level, we generated 10,000 Markov sequences per level comprising 672 stimuli per sequence. We then estimated the Kullback–Leibler divergence (KL divergence) between each example sequence and the generating source. In particular, for Level 0 sequences this was defined as

\newcommand{\bialpha}{\boldsymbol{\alpha}}\newcommand{\bibeta}{\boldsymbol{\beta}}\newcommand{\bigamma}{\boldsymbol{\gamma}}\newcommand{\bidelta}{\boldsymbol{\delta}}\newcommand{\bivarepsilon}{\boldsymbol{\varepsilon}}\newcommand{\bizeta}{\boldsymbol{\zeta}}\newcommand{\bieta}{\boldsymbol{\eta}}\newcommand{\bitheta}{\boldsymbol{\theta}}\newcommand{\biiota}{\boldsymbol{\iota}}\newcommand{\bikappa}{\boldsymbol{\kappa}}\newcommand{\bilambda}{\boldsymbol{\lambda}}\newcommand{\bimu}{\boldsymbol{\mu}}\newcommand{\binu}{\boldsymbol{\nu}}\newcommand{\bixi}{\boldsymbol{\xi}}\newcommand{\biomicron}{\boldsymbol{\micron}}\newcommand{\bipi}{\boldsymbol{\pi}}\newcommand{\birho}{\boldsymbol{\rho}}\newcommand{\bisigma}{\boldsymbol{\sigma}}\newcommand{\bitau}{\boldsymbol{\tau}}\newcommand{\biupsilon}{\boldsymbol{\upsilon}}\newcommand{\biphi}{\boldsymbol{\phi}}\newcommand{\bichi}{\boldsymbol{\chi}}\newcommand{\bipsi}{\boldsymbol{\psi}}\newcommand{\biomega}{\boldsymbol{\omega}}\[{\rm{KL}} = \sum\limits_{{\rm{target}}} Q \left( {{\rm{target}}} \right)\log {{Q\left( {{\rm{target}}} \right)} \over {P\left( {{\rm{target}}} \right)}}{\rm {,}}\]

and for Level 1 and 2 sequences it was defined as

\newcommand{\bialpha}{\boldsymbol{\alpha}}\newcommand{\bibeta}{\boldsymbol{\beta}}\newcommand{\bigamma}{\boldsymbol{\gamma}}\newcommand{\bidelta}{\boldsymbol{\delta}}\newcommand{\bivarepsilon}{\boldsymbol{\varepsilon}}\newcommand{\bizeta}{\boldsymbol{\zeta}}\newcommand{\bieta}{\boldsymbol{\eta}}\newcommand{\bitheta}{\boldsymbol{\theta}}\newcommand{\biiota}{\boldsymbol{\iota}}\newcommand{\bikappa}{\boldsymbol{\kappa}}\newcommand{\bilambda}{\boldsymbol{\lambda}}\newcommand{\bimu}{\boldsymbol{\mu}}\newcommand{\binu}{\boldsymbol{\nu}}\newcommand{\bixi}{\boldsymbol{\xi}}\newcommand{\biomicron}{\boldsymbol{\micron}}\newcommand{\bipi}{\boldsymbol{\pi}}\newcommand{\birho}{\boldsymbol{\rho}}\newcommand{\bisigma}{\boldsymbol{\sigma}}\newcommand{\bitau}{\boldsymbol{\tau}}\newcommand{\biupsilon}{\boldsymbol{\upsilon}}\newcommand{\biphi}{\boldsymbol{\phi}}\newcommand{\bichi}{\boldsymbol{\chi}}\newcommand{\bipsi}{\boldsymbol{\psi}}\newcommand{\biomega}{\boldsymbol{\omega}}\[{\rm{KL}} = \sum\limits_{{\rm{context}}} {\biggl( {Q\left( {{\rm{context}}} \right)\cdot\sum\limits_{{\rm{target}}} Q \left( {{\rm{target}}|{\rm{context}}} \right) \log {{Q\left( {{\rm{target}}|{\rm{context}}} \right)} \over {P\left( {{\rm{target}}|{\rm{context}}} \right)}}} \biggr)} {\rm {,}}\]

where *P*( ) refers to probabilities or conditional probabilities derived from the presented sequences and *Q*( ) refers to those specified by the source. We selected 50 sequences with the lowest KL divergence (i.e., these sequences closely matched the Markov model per level). The sequences presented to the participants during the experiments were selected randomly from this sequence set.

For each trial, a sequence of 8–14 stimuli appeared in the center of the screen, one at a time in a continuous stream, for 300 ms each followed by a central white fixation dot (interstimulus interval) for 500 ms (Figure 1a). This variable trial length ensured that observers maintained attention during the whole trial. Each block comprised an equal number of trials with the same number of stimuli. The end of each trial was indicated by a red-dot cue that was presented for 500 ms. Following this, all four symbols were shown in a 2 × 2 grid. The positions of test stimuli were randomized from trial to trial. Observers were asked to indicate which symbol they expected to appear following the preceding sequence by pressing a key corresponding to the location of the predicted symbol. Observers learned a stimulus–key mapping during the familiarization phase: 8, 9, 5, and 6 on the number pad corresponded to the four positions of the test stimuli—upper left, upper right, lower left, and lower right, respectively. After the observer's response, a white circle appeared on the selected stimulus for 300 ms to indicate the observer's choice, followed by a fixation dot for 150 ms (intertrial interval) before the start of the next trial. If no response was made within 2 s, a null response was recorded and the next trial started. Participants were given feedback (i.e., score in the form of a performance index; see Data analysis) at the end of each block—rather than per-trial error feedback—that motivated them to continue with training.

#### Test sessions

Test sessions were conducted at the beginning and end of Experiments 1 and 2. Pre- and posttraining test sessions comprised nine runs (i.e., three runs per level). Intermediate test sessions (i.e., test sessions after training per level) included nine runs with sequences from the trained level. Each run comprised five blocks of structured and five blocks of random sequences presented in random order (two trials per block; a total of 10 structured and 10 random trials per run). For random sequences the four symbols were presented with equal probability in a random order. Each trial comprised a sequence of 10 symbols that were presented for 250 ms each, separated by a blank interval during which a white fixation dot was presented for 250 ms. Following the sequence, a response cue (central red dot) appeared on the screen before the four test stimuli were displayed for 1.5 s. No feedback was given during the test sessions.

### Data analysis

#### Performance index

We assessed participant responses in a probabilistic manner. For each context, we computed the absolute Euclidean distance between the distribution of participant responses and the distribution of presented targets estimated across 56 trials per block:

\newcommand{\bialpha}{\boldsymbol{\alpha}}\newcommand{\bibeta}{\boldsymbol{\beta}}\newcommand{\bigamma}{\boldsymbol{\gamma}}\newcommand{\bidelta}{\boldsymbol{\delta}}\newcommand{\bivarepsilon}{\boldsymbol{\varepsilon}}\newcommand{\bizeta}{\boldsymbol{\zeta}}\newcommand{\bieta}{\boldsymbol{\eta}}\newcommand{\bitheta}{\boldsymbol{\theta}}\newcommand{\biiota}{\boldsymbol{\iota}}\newcommand{\bikappa}{\boldsymbol{\kappa}}\newcommand{\bilambda}{\boldsymbol{\lambda}}\newcommand{\bimu}{\boldsymbol{\mu}}\newcommand{\binu}{\boldsymbol{\nu}}\newcommand{\bixi}{\boldsymbol{\xi}}\newcommand{\biomicron}{\boldsymbol{\micron}}\newcommand{\bipi}{\boldsymbol{\pi}}\newcommand{\birho}{\boldsymbol{\rho}}\newcommand{\bisigma}{\boldsymbol{\sigma}}\newcommand{\bitau}{\boldsymbol{\tau}}\newcommand{\biupsilon}{\boldsymbol{\upsilon}}\newcommand{\biphi}{\boldsymbol{\phi}}\newcommand{\bichi}{\boldsymbol{\chi}}\newcommand{\bipsi}{\boldsymbol{\psi}}\newcommand{\biomega}{\boldsymbol{\omega}}\[{\rm{AbDist}}\left( {{\rm{context}}} \right){\rm{\ }} = {\rm{\ }}{\sum _{{\rm{target}}}}\left| {{{\rm{P}}_{{\rm{resp}}}}({\rm{target}}} \right|{\rm{context}}){\rm{\ }} - {\rm{\ }}{{\rm{P}}_{{\rm{pres}}}}\left( {{\rm{target}}|{\rm{context}}} \right),\]

where the sum is over targets from the symbol set A, B, C, and D. We estimate AbDist per context for each block. We quantified the minimum overlap between these two distributions by computing a Performance Index (PI) per context:

\newcommand{\bialpha}{\boldsymbol{\alpha}}\newcommand{\bibeta}{\boldsymbol{\beta}}\newcommand{\bigamma}{\boldsymbol{\gamma}}\newcommand{\bidelta}{\boldsymbol{\delta}}\newcommand{\bivarepsilon}{\boldsymbol{\varepsilon}}\newcommand{\bizeta}{\boldsymbol{\zeta}}\newcommand{\bieta}{\boldsymbol{\eta}}\newcommand{\bitheta}{\boldsymbol{\theta}}\newcommand{\biiota}{\boldsymbol{\iota}}\newcommand{\bikappa}{\boldsymbol{\kappa}}\newcommand{\bilambda}{\boldsymbol{\lambda}}\newcommand{\bimu}{\boldsymbol{\mu}}\newcommand{\binu}{\boldsymbol{\nu}}\newcommand{\bixi}{\boldsymbol{\xi}}\newcommand{\biomicron}{\boldsymbol{\micron}}\newcommand{\bipi}{\boldsymbol{\pi}}\newcommand{\birho}{\boldsymbol{\rho}}\newcommand{\bisigma}{\boldsymbol{\sigma}}\newcommand{\bitau}{\boldsymbol{\tau}}\newcommand{\biupsilon}{\boldsymbol{\upsilon}}\newcommand{\biphi}{\boldsymbol{\phi}}\newcommand{\bichi}{\boldsymbol{\chi}}\newcommand{\bipsi}{\boldsymbol{\psi}}\newcommand{\biomega}{\boldsymbol{\omega}}\[{\rm{PI}}\left( {{\rm{context}}} \right){\rm{\ }} = {\rm{\ }}{\sum _{{\rm{target}}}}{\rm{min}}\left( {{{\rm{P}}_{{\rm{resp}}}}\left( {{\rm{target}}|{\rm{context}}} \right),{\rm{\ }}{{\rm{P}}_{{\rm{pres}}}}\left( {{\rm{target}}|{\rm{context}}} \right)} \right).\]

Note that PI(context) = 1 − AbDist(context)/2. The overall performance index is then computed as the average of the performance indices across contexts, PI(context), weighted by the corresponding stationary context probabilities:

\newcommand{\bialpha}{\boldsymbol{\alpha}}\newcommand{\bibeta}{\boldsymbol{\beta}}\newcommand{\bigamma}{\boldsymbol{\gamma}}\newcommand{\bidelta}{\boldsymbol{\delta}}\newcommand{\bivarepsilon}{\boldsymbol{\varepsilon}}\newcommand{\bizeta}{\boldsymbol{\zeta}}\newcommand{\bieta}{\boldsymbol{\eta}}\newcommand{\bitheta}{\boldsymbol{\theta}}\newcommand{\biiota}{\boldsymbol{\iota}}\newcommand{\bikappa}{\boldsymbol{\kappa}}\newcommand{\bilambda}{\boldsymbol{\lambda}}\newcommand{\bimu}{\boldsymbol{\mu}}\newcommand{\binu}{\boldsymbol{\nu}}\newcommand{\bixi}{\boldsymbol{\xi}}\newcommand{\biomicron}{\boldsymbol{\micron}}\newcommand{\bipi}{\boldsymbol{\pi}}\newcommand{\birho}{\boldsymbol{\rho}}\newcommand{\bisigma}{\boldsymbol{\sigma}}\newcommand{\bitau}{\boldsymbol{\tau}}\newcommand{\biupsilon}{\boldsymbol{\upsilon}}\newcommand{\biphi}{\boldsymbol{\phi}}\newcommand{\bichi}{\boldsymbol{\chi}}\newcommand{\bipsi}{\boldsymbol{\psi}}\newcommand{\biomega}{\boldsymbol{\omega}}\[{\rm{PI\ }} = {\rm{\ }}{\sum _{{\rm{context}}}}{\rm{PI}}\left( {{\rm{context}}} \right){\rm{\ }}\cdot{\rm{\ P}}\left( {{\rm{context}}} \right).\]

To compare across different levels, we defined a normalized PI measure that quantifies participant performance relative to random guessing. We computed a random-guess baseline—that is, performance index PI_rand_—that reflects participant responses to targets with equal probability of 25% for each target per trial for Level 0 (PI_rand_ = 0.53) and equal probability for each target for a given context for Levels 1 and 2 (PI_rand_ = 0.45). To correct for differences in random-guess baselines across levels, we subtracted the random-guess baseline from the performance index (PI_normalized_ = PI − PI_rand_).

#### Strategy choice and strategy index

To quantify each observer's strategy, we compared individual participant responses to probability matching, where probability distributions are derived from the Markov models that generated the presented sequences (matching), and probability maximization, where only the single most likely outcome is allowed for each context (maximization). We used KL divergence to compare the response distribution to matching versus maximization. KL is defined as follows:

\newcommand{\bialpha}{\boldsymbol{\alpha}}\newcommand{\bibeta}{\boldsymbol{\beta}}\newcommand{\bigamma}{\boldsymbol{\gamma}}\newcommand{\bidelta}{\boldsymbol{\delta}}\newcommand{\bivarepsilon}{\boldsymbol{\varepsilon}}\newcommand{\bizeta}{\boldsymbol{\zeta}}\newcommand{\bieta}{\boldsymbol{\eta}}\newcommand{\bitheta}{\boldsymbol{\theta}}\newcommand{\biiota}{\boldsymbol{\iota}}\newcommand{\bikappa}{\boldsymbol{\kappa}}\newcommand{\bilambda}{\boldsymbol{\lambda}}\newcommand{\bimu}{\boldsymbol{\mu}}\newcommand{\binu}{\boldsymbol{\nu}}\newcommand{\bixi}{\boldsymbol{\xi}}\newcommand{\biomicron}{\boldsymbol{\micron}}\newcommand{\bipi}{\boldsymbol{\pi}}\newcommand{\birho}{\boldsymbol{\rho}}\newcommand{\bisigma}{\boldsymbol{\sigma}}\newcommand{\bitau}{\boldsymbol{\tau}}\newcommand{\biupsilon}{\boldsymbol{\upsilon}}\newcommand{\biphi}{\boldsymbol{\phi}}\newcommand{\bichi}{\boldsymbol{\chi}}\newcommand{\bipsi}{\boldsymbol{\psi}}\newcommand{\biomega}{\boldsymbol{\omega}}\[KL = \mathop \sum \limits_{target} M\left( {target} \right)log\left({{M(target)} \over {R(target)}}\right)\]

for the Level 0 model and

\newcommand{\bialpha}{\boldsymbol{\alpha}}\newcommand{\bibeta}{\boldsymbol{\beta}}\newcommand{\bigamma}{\boldsymbol{\gamma}}\newcommand{\bidelta}{\boldsymbol{\delta}}\newcommand{\bivarepsilon}{\boldsymbol{\varepsilon}}\newcommand{\bizeta}{\boldsymbol{\zeta}}\newcommand{\bieta}{\boldsymbol{\eta}}\newcommand{\bitheta}{\boldsymbol{\theta}}\newcommand{\biiota}{\boldsymbol{\iota}}\newcommand{\bikappa}{\boldsymbol{\kappa}}\newcommand{\bilambda}{\boldsymbol{\lambda}}\newcommand{\bimu}{\boldsymbol{\mu}}\newcommand{\binu}{\boldsymbol{\nu}}\newcommand{\bixi}{\boldsymbol{\xi}}\newcommand{\biomicron}{\boldsymbol{\micron}}\newcommand{\bipi}{\boldsymbol{\pi}}\newcommand{\birho}{\boldsymbol{\rho}}\newcommand{\bisigma}{\boldsymbol{\sigma}}\newcommand{\bitau}{\boldsymbol{\tau}}\newcommand{\biupsilon}{\boldsymbol{\upsilon}}\newcommand{\biphi}{\boldsymbol{\phi}}\newcommand{\bichi}{\boldsymbol{\chi}}\newcommand{\bipsi}{\boldsymbol{\psi}}\newcommand{\biomega}{\boldsymbol{\omega}}\[KL = \mathop \sum \limits_{context} M\left( {context} \right)\mathop \sum \limits_{target} M\left( {target|context} \right)log\left({{M(target|context)} \over {R\left( {target} \right)|context}}\right)\]

for the Levels 1 and 2 model, where *R*( ) and *M*( ) denote the probability distribution or conditional probability distribution derived from the human responses and probability matching versus maximization respectively, across all the conditions.

We quantified the difference between the KL divergence from maximization and matching to the response-based distribution, respectively. We refer to this quantity as *strategy choice*, indicated by ΔKL(maximization, matching). We updated the strategy choice per trial and averaged across blocks, resulting in a strategy curve across training for each individual participant. We then derived an individual strategy index by calculating the integral of each participant's strategy curve and subtracting it from the integral of the exact matching curve, as defined by matching across training. We defined the integral-curve difference between individual strategy and exact matching as the individual strategy index.

## Results

### Experiment 1: Behavioral performance

Previous studies have compared learning of different spatiotemporal contingencies in separate experiments across different participant groups (Fiser & Aslin, 2002a, 2005). Here, to investigate whether individuals extract changes in structure, we presented the same participants with sequences that changed in structure unbeknownst to them (Figure 1a). We parameterized structure complexity based on the memory order of the Markov models used to generate the sequences—that is, the degree to which the presentation of a symbol depended on the history of previously presented symbols (Figure 1b). We first presented participants with simple zero-order sequences (Level 0), followed by more complex first- and second-order sequences (Level 1, Level 2), as previous work has shown that temporal dependencies are more difficult to learn as their length increases (van den Bos & Poletiek, 2008) and training with simple dependencies may facilitate learning of more complex contingencies (Antoniou, Ettlinger, & Wong, 2016). Zero-order sequences (Level 0) were contextless—that is, the presentation of each symbol depended only on the probability of occurrence of each symbol. First- and second-order sequences were governed by context-based statistics—that is, the presentation of a particular symbol was conditionally dependent on the previously presented symbols (i.e., context length of 1 or 2). Participants were presented with first-order (Level 1: context length of one stimulus) followed by variable-order (Level 2: context length of one or two stimuli) context–target contingencies. We measured participant performance in the prediction task before and after training.

As the sequences we employed were stochastic, we developed a probabilistic measure to assess participants' performance in the prediction task. Specifically, we computed a performance index (PI) that indicates how closely the distribution of participant responses matched the probability distribution of the presented symbols. This is preferable to a simple measure of accuracy because the probabilistic nature of the sequences means that the correct upcoming symbol is not uniquely specified; thus, designating a particular choice as correct or incorrect is often arbitrary.

**Figure 2:**
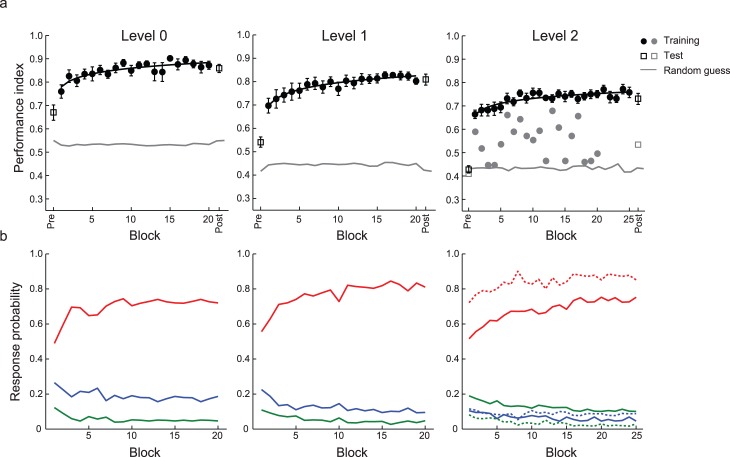
Experiment 1: Behavioral performance. (a) Performance index for Group 0 (n = 19) across training (solid circles) blocks, pretraining test (Pre: open squares), and posttraining test (Post: open squares). The performance index expresses the absolute distance (proportion overlap) between the distribution of participant responses and the distribution of presented targets. Overall performance index is calculated as the weighted average across context probabilities. Data are fitted for participants who improved during training (black circles). Data are also shown for one participant who did not improve during training (Level 2, gray symbols). Error bars show standard error of the mean. (b) Response probabilities for individual targets (Level 0) or conditional probabilities of context–target contingencies (Levels 1 and 2) across training blocks. Red lines indicate targets or context–target contingencies with the highest (conditional) probability (i.e., 0.72 for Level 0 and 0.8 for Levels 1 and 2), blue lines indicate the second-highest (conditional) probabilities (i.e., 0.18 for Level 0 and 0.2 for Levels 1 and 2), and green lines indicate targets or context–target contingencies that appear rarely (i.e., 0.05) or not at all. For Level 2, first- and second-order contexts are presented separately (dashed vs. solid lines).

Our results showed fast learning initially (i.e., enhanced performance in the first two training blocks compared to the pretraining test) that was followed by further improvement during the rest of the training (Figure 2a). This is consistent with the time course demonstrated by previous perceptual-learning studies (Karni & Sagi, 1993). Comparing normalized performance (i.e., after subtracting random guessing) before and after training showed that participants were able to learn the presented sequences (only one participant showed less than 10% improvement after four training sessions for Level 2). A repeated-measures ANOVA with session (pre-, posttest) and complexity level (0, 1, 2) as factors showed significant main effects of session, *F*(1, 18) = 145.8, *p* < 0.001, and level, *F*(1, 18) = 19.0, *p* < 0.001, consistent with enhanced performance after training and increasing task difficulty for higher order sequences. Further, the lack of a significant interaction between session and level, *F*(2, 36) = 2.40, *p* = 0.106, suggests similar improvement across levels.

The learning functions in Figure 2a highlight that performance improves through training. Next we directly assessed how well participants were able to extract structures that were predictive of upcoming events. Figure 2b shows that the participants' ability to extract the most frequently presented symbols (Level 0) or context–target contingencies (Levels 1 and 2) improved with training across levels. When participants were presented with sequences of variable context length (Level 2), they maintained good performance for the first-order contingencies and also improved in extracting second-order contingencies.

Finally, we asked whether these learning effects were specific to the trained sequences. First, we contrasted performance accuracy on structured versus random sequences before and after training sessions. We found significant interactions between session and sequence, indicative of effects specific to the structured sequences—Level 0: *F*(1, 18) = 9.17, *p* = 0.007; Level 1: *F*(1, 18) = 83.8, *p* < 0.001; Level 2: *F*(1, 18) = 61.7, *p* < 0.001. Second, we conducted a no-training control experiment. Participants (*n* = 11) were tested with structured sequences in two sessions, but they did not receive training between sessions. Our results showed no significant main effect of session, *F*(1, 10) = 0.12, *p* = 0.736, or level, *F*(1, 10) = 1.84, *p* = 0.205, nor a significant interaction between session and level, *F*(1, 10) = 1.16, *p* = 0.308, indicating that improvements were specific to trained sequences rather than a result of repeated exposure during the pre- and posttraining sessions.

### Response tracking

To quantify our results, we tracked the participants' responses across trials using a weighted combination (i.e., mixture) of Markov processes (i.e., zero-, first-, second-order). Previous work has used a Hebbian process to account for perceptual learning without explicit feedback (Liu, Lu, & Dosher, 2010; Petrov, Dosher, & Lu, 2005, 2006). For our purposes, however, capturing the dynamics of participants' responses as they learn to condition their responses on higher order statistics is difficult for a Hebbian process, due to the limited discrete data (i.e., one response per trial) during the learning process. Following previous work on the learning of visual statistics (Droll, Abbey, & Eckstein, 2009; Eckstein, Abbey, Pham, & Shimozaki, 2004), we used a Bayesian process to adjust the mixture coefficient weights assigned to these component Markov processes during training (Supplementary Material). In particular, we extracted changes in participants' responses over time that relate to the rule used to generate the sequences—that is, memory or context length (e.g., the current target depends on the last symbol or the last two symbols)—and to the contingencies between individual stimuli in the sequence (e.g., last stimulus was A, so next is likely to be B).

### Extracting context length from participants' responses

**Figure 3:**
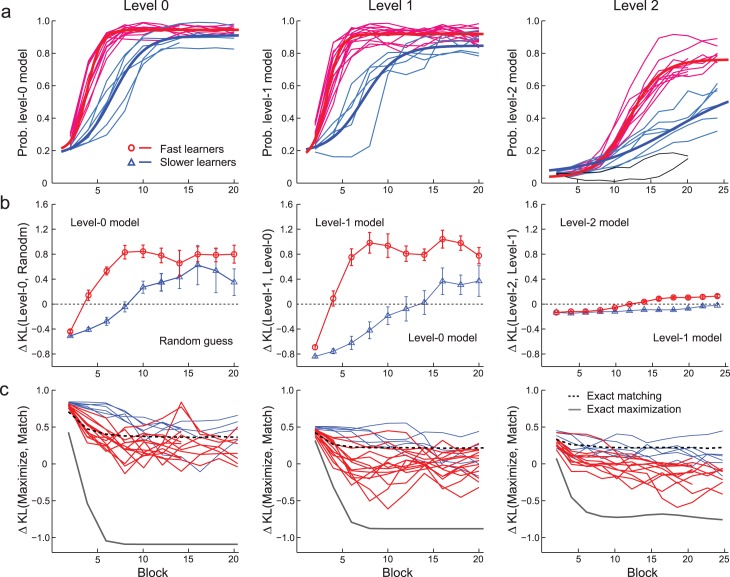
Experiment 1: Response tracking. (a) Functional clustering analysis (Group 0) showed two data clusters, indicated in red (Level 0: n = 13, Level 1: n = 14, Level 2: n = 11) versus blue (Level 0: n = 6, Level 1: n = 5, Level 2: n = 6). Mixture coefficient curves are shown for each individual participant; bold curves indicate sigmoid fits to each cluster. Data are also shown for two participants (black lines) who showed less than a 25% probability of extracting the correct context length at the end of training. (b) Learning predictive probabilities. ΔKL curves between the predictive mixture model for each level and baseline models across training blocks. ΔKL values above zero indicate that the participant responses approximated the Markov model that generated the sequences. Average data are shown per participant cluster (i.e., red vs. blue). Note: The smaller ΔKL values and error bars for Level 2 reflect small differences between Level 1 and Level 2 models; yet fast learners show higher values than zero, indicating that they are able to learn second-order context–target contingencies. Error bars show the standard error of the mean. (c) Strategy choice, as indicated by comparing (ΔKL) matching versus maximization for each participant per cluster (i.e., red vs. blue).

First, we asked whether participants were able to extract the correct context length during training. In particular, a significant increase in the mixture coefficient for a given Markov order (e.g., Level 1) provides an indication that participants use a given memory length (e.g., context length 1) when responding. As the participants learned, we dynamically tracked whether and when the memory (context length) in their responses changed. In particular, we traced the evolution of the coefficients of the individual mixture components across training blocks. Mixture coefficient curves for individual participants followed a sigmoid shape, indicating changes in the context length extracted by the observers during training; we refer to these curves as *learning curves*. This analysis (Figure 3a) revealed that most participants became better at extracting the correct context length during training, except two participants (gray lines for Level 2 in Figure 3a) who showed less than a 25% probability of selecting the correct context length. Further, comparing learning rate—as determined by the sigmoid mixture coefficient curves—across levels (0, 1, and 2) showed significantly slower learning rates for higher order than simpler sequences, *F*(2, 49) = 23.7, *p* < 0.001.

A notable feature of the learning curves in Figure 3a is the variability in learning rates between different participants: Some individuals extracted the correct context length earlier in the training than others. To characterize prototypical learning profiles, we performed a functional clustering analysis of the learning curves (Supplementary Material). We found that two clusters were adequate to capture the individual variability in the data (Supplementary Figure S1). Given the apparent difference between participant groups in the speed of extracting the correct context length, we refer to these clusters as fast and slower learners. Supplementary Figure S2 shows differences in the learning rate of the more probable contingencies between the two clusters, confirming that some learners extracted the behaviorally relevant statistics faster than others.

We took a number of steps to validate our response-tracking analysis in a controlled manner. As a first step, we applied this analysis to random responses. We found no evolution of the coefficients of the individual mixture components, suggesting that the changes revealed using the participants' data do not simply reflect the dynamics of parameter initialization. We also tested our response-tracking analysis on responses generated by a synthetic learner (Supplementary Material), controlling for key parameters (learning rate and memory-order transition point). We varied the synthetic learner's parameters and recorded the sequence of predictions it made. This test showed that we could recover the key parameters that determined the synthetic learner's predictions (Supplementary Figure S3).

### Extracting predictive contingencies from participants' responses

For individuals to succeed in the prediction task, they needed to extract not only the appropriate context length but also the correct conditional probabilities (i.e., context–target contingencies). To capture the dynamics of learning predictive contingencies, we sought to quantify the relationship between the participants' responses and the Markov models used to generate stimulus sequences. For each Markov order level, we considered two alternative models: the correct model order (e.g., Level 1 choices for Level 1 sequences) or a lower order approximation based on the previously trained sequence level (e.g., Level 0 choices for Level 1 sequences). We initially favored the lower order approximation to prevent emulating lower order structure using a higher order model. Using a Bayesian updating process, we obtained evidence that allowed us to discern whether responses were governed by a lower or a higher order process. We quantified how close participants' behavior was to a particular model using the Kullback–Leibler (KL) divergence statistic. We then contrasted KL statistics (i.e., slope of ΔKL learning curves) to test which model the participants' responses approximated (Figure 3b). A two-way ANOVA showed a significant interaction between complexity level (0, 1, 2) and cluster (fast vs. slower learners), *F*(2, 49) = 3.90, *p* = 0.027, suggesting that individuals who extracted the correct context length early in the training also learned the appropriate context–target contingencies. Further, we observed a main effect of level—fast learners: *F*(2, 49) = 39.0, *p* < 0.001; slower learners: *F*(2, 49) = 4.90, *p* = 0.012—suggesting that learning the correct predictive contingencies was more difficult for higher order sequences.

Previous work (Jensen, Boley, Gini, & Schrater, 2005) has demonstrated that temporal structure can be extracted without an explicit representation of the underlying model based on computing the entropy of excerpts from temporal sequences. We implemented an entropy-based approach and showed that it could recover first- and second-order contexts from the participant responses (Supplementary Figure S4). However, we found that this approach was limited in tracking the learning dynamics, as it required more trials to extract learning strategies from participant responses (i.e., there were insufficient participant responses to reliably estimate entropy in the first 10 blocks of trials).

### Strategies for probability learning: Matching versus maximization

As the Markov models that generated stimulus sequences were stochastic, participants needed to learn the probabilities of different outcomes to succeed in the prediction task. Motivated by previous work on decision making in the context of cognitive (Shanks, Tunney, & McCarthy, 2002) and sensorimotor tasks (Acerbi, Vijayakumar, & Wolpert, 2014; Eckstein et al., 2013; Murray, Patel, & Yee, 2015), we formulated two possible strategies for making predictions. First, participants might use *probability maximization*, whereby they would always select the most probable outcome in a particular context. Alternatively, they might learn the relative probabilities of each symbol—for example, *p*(A) = 0.18, *p*(B) = 0.72, *p*(C) = 0.05, *p*(D) = 0.05—and respond so as to reproduce this distribution, a strategy referred to as *probability matching*.

To quantify participants' strategies across training, we computed a strategy index that indicates each participant's preference (on a continuous scale) for responding using probability matching versus maximization (Figure 3c). We found that for Level 0 sequences, participants adopted a strategy that was closer to probability matching than maximization, suggesting that they solved the task by memorizing the frequency with which each symbol occurred. However, for Levels 1 and 2 they shifted toward maximization. Comparing individual strategy across levels and participant clusters showed a significant main effect of complexity level, *F*(2, 49) = 12.2, *p* < 0.001, suggesting that participants' strategies shifted closer to maximization for higher order sequences. Further, a significant main effect of cluster, *F*(1, 49) = 60.9, *p* < 0.001, indicates that fast learners who extracted the correct context length early in training deviated from matching and adopted a strategy closer to maximization. The lack of a significant interaction between cluster and level, *F*(2, 18) = 0.025, *p* = 0.915, suggests that each cluster of participants adopted a similar strategy across levels (i.e., closer to maximization for fast than for slower learners).

Despite greater maximization at higher complexities, we note that participants did not achieve optimal maximization performance (Figure 3c). Maximization is typically observed under supervised or reinforcement learning paradigms (Shanks et al., 2002), so it is perhaps not surprising that our participants did not achieve exact maximization, as trial-by-trial feedback was not provided. Moreover, the tendency for participants to respond using probability matching may be higher when individual elements are clearly discriminable (i.e., our symbols) but nevertheless ambiguous because different processes can give rise to similar sequences of symbols (as in our sequence-generation process; Murray et al., 2015). Our findings are consistent with previous studies showing that participants adopt a strategy closer to matching when learning a simple probabilistic task in the absence of trial-by-trial feedback (Shanks et al., 2002). However, for more complex probabilistic tasks, participants weight their responses toward the most-likely outcome (i.e., adopt a strategy closer to maximization) after training (Lagnado, Newell, Kahan, & Shanks, 2006).

### Experiment 2: Behavioral performance

We next asked whether learning of simple structures facilitates subsequent learning of complex structures. In Experiment 2, we tested two additional participant groups who started training from Level 1 (Group 1) or Level 2 (Group 2) rather than Level 0. We then compared performance in Groups 1 and 2 with performance by participants who trained on all three levels (i.e., Experiment 1, Group 0).

**Figure 4:**
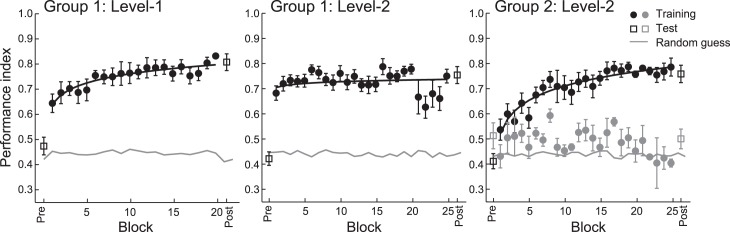
Experiment 2: Behavioral performance. Data for Group 1 (n = 8; Levels 1 and 2) and Group 2 (n = 12; Level 2). Performance index is shown across training (solid circles) blocks, pretraining test (Pre: open squares), and posttraining test (Post: open squares). Fitted data are shown for participants who improved during training (black circles). Data are also shown for participants (n = 4) in Group 2 who did not improve during training (Level 2, gray symbols). Error bars show standard error of the mean.

Group 1 participants (*n* = 8) were first trained on Level 1 and then Level 2, but not Level 0. The results from this group (Figure 4) were similar to the results from Experiment 1. In particular, comparing performance between Group 0 and Group 1 (three-way mixed ANOVA) showed significant effects of session (pre vs. post), *F*(1, 25) = 191.3, *p* < 0.001, and complexity level (1 vs. 2), *F*(1, 25) = 25.9, *p* < 0.001, but no significant effect of group, *F*(1, 25) = 0.253, *p* = 0.619, nor any significant interactions: session, level, and group, *F*(1, 25) = 0.311, *p* = 0.582; session and group, *F*(1, 25) = 2.22, *p* = 0.149; level and group, *F*(1, 25) = 1.15, *p* = 0.293. Further, comparing initial training performance (mean of first two training blocks) between the two groups did not show a significant group effect, *F*(1, 25) = 0.106, *p* = 0.747, suggesting that training with zero-order sequences does not facilitate the learning of higher order sequences.

In contrast, extracting higher order structures proved to be more difficult for Group 2 participants (*n* = 12), who did not have prior experience with zero- or first-order sequences (Figure 4). In particular, eight of 12 participants improved significantly in the task during training, while the rest of the participants showed less than 10% improvement. A mixed ANOVA comparing training session (start, end of training) and group (0, 1, 2) showed a significant interaction between the two, *F*(2, 31) = 4.41, *p* = 0.021. In particular, there was a significant difference between groups in performance at the start, *F*(2, 31) = 5.14, *p* = 0.012, but not the end of training, *F*(2, 31) = 0.893, *p* = 0.420. To investigate this difference further, we compared performance on the second-order contexts only (i.e., excluding first-order contexts in Level 2) between groups. There was a significant interaction between session and group, *F*(2, 31) = 10.52, *p* < 0.001, and a significant difference between groups in performance at the start, *F*(2, 32) = 5.05, *p* = 0.013, but not at the end of training, *F*(2, 32) = 1.75, *p* = 0.191. Post hoc comparisons showed significantly higher performance indices in the prediction task for second-order contexts in Group 0 and Group 1 than in Group 2—Group 0 versus Group 2: *p* = 0.023; Group 1 versus Group 2: *p* = 0.009.

Taken together, these results suggest that learning first-order sequences facilitates learning of higher order sequences. In contrast, learning frequency statistics does not facilitate performance in learning higher order sequences. Further, fast learners in Experiment 2 extracted the correct context length and context–target contingencies early in training and deviated from matching toward maximization (Supplementary Figure S5). In particular, fast learners extracted second-order contexts earlier than slower learners, who continued to rely on first-order contexts (Supplementary Figure S6).

### Tracking individual strategy across levels

**Figure 5:**
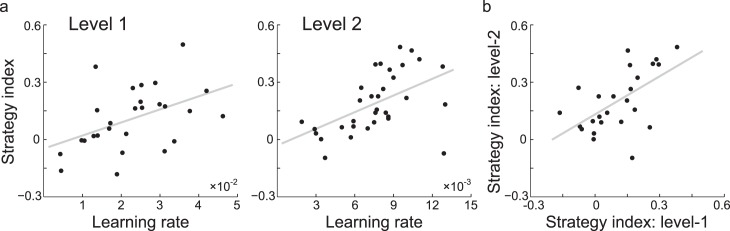
Strategies for learning context-based statistics. (a) Correlations of individual strategy index and learning rate for participants who improved at both Levels 1 and 2 during training in Group 0 and Group 1. (b) Correlation of individual strategy index between Level 1 and Level 2 for participants trained in Group 0 and Group 1. Negative strategy-index values indicate a strategy closer to matching, while positive values indicate a strategy closer to maximization.

Combining data across experiments, we asked how individual strategy relates to learning performance (i.e., learning rate). Significant correlations (Figure 5a) between participants' learning rate and strategy index—Level 1 (*n* = 27): *R* = 0.461, *p* = 0.016; Level 2 (*n* = 33): *R* = 0.519, *p* = 0.002—indicate that participants who extracted the correct context length early in the training adopt a strategy closer to maximization. These results suggest that fast learning relates to selecting the most probable outcome when learning context–target contingencies. We then asked how the participants' strategies developed during training across levels. Correlating individual strategy index across Levels 1 and 2 (Figure 5b) showed that participants' strategy was highly correlated (*R* = 0.489, *p* = 0.0131) across Levels 1 and 2 (*n* = 25 from Groups 0 and 1). These results suggest that participants mostly retained the same strategy across levels of complexity (i.e., from first- to second-order sequences).

## Discussion

Here we ask how individuals adapt to changes in the environment's statistics to make predictions about future events. In particular, we sought to characterize the dynamics of learning temporal structures that change in their complexity. We tracked each participant's responses across trials and tested whether and when participants extract the structure that governs sequences of unfamiliar symbols. This enabled us to provide the following four main advances in understanding the dynamics of human statistical learning.

First, we show that participants adapt to the environment's statistics: They extract behaviorally relevant structures from temporal sequences that change in their complexity to make predictions about upcoming events. Further, they benefit from previous exposure to lower order statistics (i.e., first-order sequences) when learning higher order structures. Previous studies (Fiser & Aslin, 2002a, 2005) have shown that humans are able to extract complex spatiotemporal statistics (e.g., joint vs. conditional probability statistics). These statistics are typically manipulated in separate short-lasting experiments and tested across separate groups of individuals. Here, we test how the same individuals extract structures that change in their complexity, simulating more naturalistic situations that require extracting a range of patterns from simple repetition to probabilistic combinations. Our response-tracking approach allows us to monitor whether and when individuals shift from learning simple to complex structures, as the complexity of the presented sequences changed unbeknownst to them. Our findings demonstrate that individuals extract the behaviorally relevant context length and context–target contingencies that correspond to the structure of the presented sequences.

Second, our response-tracking approach allowed us to extract prototypical patterns of learning dynamics. We demonstrate that fast learners succeeded in identifying the correct statistical structure early in the training. Interestingly, when learning complex structures, fast learners extracted higher order contexts and adopted a learning strategy closer to maximization (i.e., extracted the most probable target per context) earlier in the training. Previous work has tested the role of matching versus maximization strategies in perceptual decision making (Acerbi et al., 2014; Eckstein et al., 2013; Murray et al., 2015) and reward-based learning (Shanks et al., 2002): Observers may distribute their choice responses so as to match the underlying input statistics versus maximize their reward by selecting the most frequently rewarded outcome in each trial. Here, we test these strategies in the context of statistical learning. We show that fast learners tend to use a strategy closer to maximization, suggesting that there may be a benefit to extracting the most probable target per context rather than attempting to learn all statistical dependencies. Further, our findings are consistent with studies suggesting that previous experience shapes the selection of decision strategies (Fulvio, Green, & Schrater, 2014; Rieskamp & Otto, 2006).

Third, we ask whether learning temporal structures occurs in an incidental manner through exposure to regularities or whether it involves explicit knowledge of the underlying sequence structure. Previous studies have suggested that learning of regularities may occur implicitly (i.e., by mere exposure rather than external feedback) in a range of tasks: visuomotor sequence learning (Nissen & Bullemer, 1987), artificial grammar learning (Reber, 1967), probabilistic category learning (Knowlton, Squire, & Gluck, 1994), and contextual cue learning (Chun & Jiang, 1998). Most studies have focused on implicit measures of sequence learning, such as familiarity judgments or reaction times (for a review, see Schwarb & Schumacher, 2012). In contrast, our paradigm allows us to directly test whether exposure to temporal sequences facilitates observers' ability to explicitly predict the identity of the next stimulus in a sequence. Our experimental design makes it unlikely that the participants memorized specific stimulus positions or the full sequences. Further, participants were exposed to the sequences without trial-by-trial feedback, but were given block feedback about their performance that motivated them to continue with training. A control experiment during which the participants were not given any feedback showed similar results to our main experiment (Supplementary Figure S7), suggesting that it is unlikely that the block feedback facilitated explicit sequence memorization. Yet it is possible that making an explicit prediction about the identity of the test stimulus made the participants aware of the dependencies between the stimuli presented in the sequence. During debriefing, most participants reported some predictive sequence structures (i.e., high-probability symbols or context–target combinations). Thus, it is possible that prolonged exposure to probabilistic structures (i.e., multiple sessions in contrast to single-exposure sessions typically used in statistical-learning studies) in combination with prediction judgments (Dale, Duran, & Morehead, 2012) may evoke some explicit knowledge of temporal structures, in contrast to implicit measures of anticipation typically used in statistical-learning studies.

Finally, previous work has discussed a range of possible representations that are formed during statistical learning. This has mainly focused on deriving generative structure from the stimulus space (for a review, see Dehaene, Meyniel, Wacongne, Wang, & Pallier, 2015) and implicated a range of representations from learning stimulus associations and transitional probabilities to sequence chunks (i.e., statistical contingencies) and abstract rules (Aslin & Newport, 2012; Fiser, Berkes, Orbán, & Lengyel, 2010; Opitz, 2010; Orbán, Fiser, Aslin, & Lengyel, 2008; Reber, 1967). In the context of our task, extracting the sequence context length may relate to rule-based learning, while learning behaviorally relevant contingencies may relate to chunk learning. Further, this range of processes parallels the distinctions between model-free and model-based learning by exploring new strategies versus exploiting previously learned associations in the context of reward-based learning (Dayan & Niv, 2008; Koechlin, 2014). However, distinguishing between these accounts in the context of statistical learning is complicated by task setting and complexity (Franco & Destrebecqz, 2012; Pothos, 2007). Here we take a different perspective: To understand the dynamics of human behavior, we track human responses during mere exposure to temporal sequences that change in their structure, simulating interactions in naturalistic settings that vary in context and complexity. We show that learning predictive statistics proceeds without explicit trial-by-trial feedback and relates to individual strategy in extracting behaviorally relevant structure from sequences of events.

In sum, our findings provide evidence that successful learning of complex structures relies on extracting behaviorally relevant statistics that are predictive of upcoming events. This learning of predictive structures relates to individual decision strategy: Faster learning of complex structures relates to selecting the most probable outcomes in a given context rather than learning the exact sequence statistics, providing evidence for an alternate route to learning. In future work, it would be interesting to investigate whether these strategies are specific to the sensory input modality or mediate domain-general learning of temporal structure (Nastase, Iacovella, & Hasson, 2014). Recent work has provided evidence for statistical learning within and across different sensory modalities (vision, audition, touch; Conway & Christiansen, 2005; Mitchel & Weiss, 2011), suggesting that statistical learning is implemented by domain-general principles that are subject to modality-specific constraints (Frost, Armstrong, Siegelman, & Christiansen, 2015). For example, in vision statistical learning has been mainly demonstrated by extracting spatial relations, while in audition by extracting temporal regularities. Learning predictive statistics across modalities is critical not only for sensorimotor interactions with the environment but also higher cognitive functions that involve complex structures, such as action organization, music comprehension, and language learning (Conway & Christiansen, 2001; Dehaene et al., 2015; Fitch & Martins, 2014; Frost et al., 2015). Finally, it would be interesting to investigate the developmental time course of learning predictive statistics. Previous work has provided evidence for statistical learning from infancy to older age (for a review, see Krogh, Vlach, & Johnson, 2012) in both vision (e.g., Bulf, Johnson, & Valenza, 2011; Fiser & Aslin, 2001, 2002a, 2002b; Kirkham, Slemmer, & Johnson, 2002; Kirkham, Slemmer, Richardson, & Johnson, 2007) and audition (e.g., Pelucchi, Hay, & Saffran, 2009; Saffran et al., 1999; Saffran, Aslin, & Newport, 1996; Saffran, Newport, & Aslin, 1996). Further, it has been suggested that while learning probabilities is achieved early in life, learning meaningful statistical patterns develops later in adolescence (Amso & Davidow, 2012; Janacsek, Fiser, & Nemeth, 2012). This may relate to the suggestion that young children maximize, while matching develops later in life (Kam & Newport, 2009; Stevenson & Weir, 1959; Weir, 1964). Future work on the brain mechanisms of learning predictive statistics may explore the development of common brain routes to structure learning across domains of perceptual and cognitive expertise.

## Supplementary Material

Supplement 1
