# Supplementary material for: Learning predictive statistics from temporal sequences: Dynamics and strategies
Source: J Vis. 2017 Oct 2;17(12):1. doi: 10.1167/17.12.1 (PMC5627678; doi:10.1167/17.12.1)
Supplement: Supplement 1 [file jovi-17-12-02_s02.pdf]

Supplementary Material

Response-tracking analysis

To track human predictions as they evolve over time, we extracted changes in performance during training that relate to: (1) extracting the memory order that governs the sequences (i.e., identifying the context length); and (2) extracting context-target contingencies to generate a prediction about the next stimulus given the current context. We refer to these analyses as context-length and predictive contingency, respectively.

First, we describe the context length modeling, which considers participant's responses as a weighted combination of multiple Markov processes. This modelling approach enables us to track participants' learning in a unified framework. When participants are first exposed to the sequences, we reason that their responses will be driven by random guesses, which corresponds to a special setting of the zero-order (memory-less) Markov model. We reason that participants' responses will be later refined after having observed that some symbols are presented more frequently than others in a given context. At trial t, our model tracks the context-length learning using three components corresponding to no memory (zero-order Markov model $\mathcal{M}_t^0$ using empty context $\emptyset$ of zero length), shallow memory (first-order Markov model $\mathcal{M}_t^1$ using context $\mathcal{C}_t^1$ of length one) and deeper memory (variable length Markov model of maximum order two $\mathcal{M}_t^2$ using context $\mathcal{C}_t^2$ of length of one or two):

$$\mathcal{M}_t = \sum_{k\in\{0,1,2\}} w_t^k \cdot \mathcal{M}_t^k, \quad \text{subject to} \quad \sum_k w_t^k = 1 \ \text{ with } \ w_t^k \geq 0, \tag{1}$$

where w_t^k is the mixture coefficient at trial t of the component model $\mathcal{M}_t^k$ and represents the probability of using $\mathcal{M}_t^k$ for prediction at trial t. The mixture coefficients w_t^k can be thought of as expressing the "strength" at trial t of individual components $\mathcal{M}_t^k$ in the overall mixture model. By tracking the mixture coefficients over trials, we dynamically assess whether participants' responses can be accounted for by the different Markov structures. Note that in the previous sections, we use i to index consecutive symbols in a Markov sequence or, equivalently, stimuli in a continuous stimulus stream. Here, t is used to index consecutive trials which consist of 8-14 stimuli each.

Given the history of last seen symbols, model (1) gives the following probability to a target symbol s:

$$P_t(s|\{\emptyset, \mathcal{C}_t^1, \mathcal{C}_t^2\}) = w_t^0 \cdot P_t^0(s|\emptyset) + w_t^1 \cdot P_t^1(s|\mathcal{C}_t^1) + w_t^2 \cdot P_t^2(s|\mathcal{C}_t^2). \tag{2}$$

The predictive contingency distribution allows us to quantify how the participants' responses compare to the context-conditional probabilities of the generating Markov model.

To track a participant's responses over time, mixture coefficients as well as the mixture components themselves are updated after each response. We calculate whether the participant's response is more likely to be driven by a particular mixture component (e.g. Markov model of order one), and update the weight for the components accordingly. This updating is implemented in Bayesian terms: given the participant's response at trial t, each mixture coefficient w_{t-1}^k is updated proportionally to both the current coefficient value ('strength' of $\mathcal{M}_{t-1}^k$) and the

likelihood of $\mathcal{M}_{t-1}^k$ given the participant's response. Considering w_{t-1}^k and w_t^k as the prior and posterior for the model order k, Bayesian update of w_t^k reads:

$$W_t^k = \frac{w_{t-1}^k \cdot P_{t-1}^k(\hat{s}_t|\mathcal{C}_t^k)}{\sum_{k'} w_{t-1}^{k'} \cdot P_{t-1}^{k'}(\hat{s}_t|\mathcal{C}_t^{k'})}. \quad (3)$$

Similarly, by applying the Bayes rule, the context-conditional predictive distribution is obtained:

$$\mathcal{P}_t^k(s|\mathcal{C}_t^k) = \frac{\delta_{s,\hat{s}_t} \cdot P_{t-1}^k(s|\mathcal{C}_t^k)}{\sum_{s'} \delta_{s',\hat{s}_t} \cdot P_{t-1}^k(s'|\mathcal{C}_t^k)}. \quad (4)$$

Here we introduce a noise model δ representing uncertainty in the participant's prediction. If we assumed that the participant is certain about their prediction, δ would be a noise-less model (delta-function),

$$\delta_{s,\hat{s}_t} = \begin{cases} 1 & \text{if } s = \hat{s}_t \\ 0 & \text{if } s \neq \hat{s}_t. \end{cases}$$

As the participants learn during the sequence presentation, we expect that modeling of the participant's responses changes smoothly over time. This is represented by a partial adaptation of the modeling parameters towards the ideal Bayesian updates (3) and (4):

$$w_t^k = w_{t-1}^k + \eta_w \cdot \left(W_k^t - w_{t-1}^k\right) \quad (5)$$

and

$$P_t^k(s|\mathcal{C}_t^k) = P_{t-1}^k(s|\mathcal{C}_t^k) + \eta_p \cdot w_t^k \cdot \left(\mathcal{P}_t^k(s|\mathcal{C}_t^k) - P_{t-1}^k(s|\mathcal{C}_t^k)\right), \quad (6)$$

where $0 < \eta_p < 1$ and $0 < \eta_w < 1$. Here we introduced tuning parameters η_p, η_w (i.e. the adaptation rate) to calibrate the response-tracking modeling and ensure smooth learning curves. The case of $\eta_p = \eta_w = 1$ corresponds to ideal Bayesian updating. From the learning dynamics point of view, for $\eta_p, \eta_w < 1$, the change of $\mathcal{M}_t$ over t is effectively smoothed. In our study, we assume the adaptation rates attain the same value, $\eta_p = \eta_w = \eta$, and set η to 0.04. We tested whether the results of our modeling change with different adaptation parameters η. The principal results remain unchanged with a high tolerance to the tuning parameter (η in the range from 0.03 to 0.1), validating the robustness of our modeling approach.

We hypothesize three likely sequence structures (i.e. level-0, level-1 and level-2) and track whether these structures account for the participants' responses. It should be noted that we introduced a special structure for level-0 data, where participants' responses are driven by target probability, rather than context-target contingencies. Unlike the general three-component modeling described above, for level-0 we only employ two mixture components related to zero-order memory: a fixed random guess mixture component (all 4 symbols have equal probability of 25%) and a flexible memory-free process where the probabilities of the 4 symbols are treated as free variables and can be updated based on participants' responses.

To initialize the response-tracking modeling, we imposed a prior reflecting a possible memory structure used by the participants at the start of training. We initialized the mixture coefficients controlling the degree of a participant's initial preference for one mixture component over other(s) as follows:

- At *level-0* we monitor whether the participants switch from fixed random guess to an appropriate level-0 model. As the participants' performance is initially guided by random guesses, we set the initial coefficient of random guess and flexible level-0 model to w and $1 - w$, respectively. We give a clear preference to random guesses by setting w to 0.8.

- At *level-1* we monitor how the participants switch from a level-0 to a level-1 model. To reflect the participants' initial preference for a simpler model, we set the initial coefficient of level-0 and level-1 model to $w = 0.8$ and $1 - w = 0.2$, respectively.

- For *level-2* preceded by training at level-1 (Group 0 and 1), we set the initial coefficients as follows: As the participants had learnt level-1 sequences, we set the coefficient w of the level-1 model to $w = 0.8$. The remaining small weight $1 - w = 0.2$ is distributed among the level 0 and 2 in the proportion $\frac{2}{3}$ and $\frac{1}{3}$, respectively, reflecting the intuition that the more complex level-2 model is a-priori less likely. Hence the initial coefficients for level-0, level-1, and level-2 models are set to 0.13, 0.8 and 0.07, respectively. When participants perform the task at level-2 without preceding training at level-1 (Group 2), the coefficients are set to $w = 0.8$, $\frac{2}{3} \cdot (1 - w)$, and $\frac{1}{3} \cdot (1 - w)$, i.e. 0.8, 0.13, 0.07, respectively. i.e., the level 0 model is assumed to be naturally preferred.

Context-length analysis

When using the dynamic mixture modeling approach (1)–(6) to the response data from a single participant, we obtain time series of mixture coefficients $\{w_t^k\}$ and the context-target contingencies $\{P_t^k\}$, $k = 0, 1, 2$, indexed by the trial index t. The initial settings described above may produce a small systematic variation on the mixture coefficients w_t^k. Therefore, to deal with uncertainty about the participants' initial preferences, we vary the initial mixture coefficients by setting w to a range of 5 values: 0.7, 0.75, 0.8, 0.85, 0.9. For every data level, participant and mixture component k, we then average the resulting 5 trajectories of w_t^k into a single mixture coefficient curve w_t^k. We followed the same method to produce the averaged context-target contingency curves $\{P_t^k\}$.

For each participant and data level k_0, we are interested in the dynamics across all training blocks for that level k_0 (model order used to generate the data). We denote the series $\{w_t^{k_0}\}$ by $\{\widetilde{w}_t\}$. The $\widetilde{w}_t$ curves thus represent the dynamics of participants' learning of the correct memory structure when producing predictions for the target stimuli. As the $\widetilde{w}_t$ curves have a sigmoid structure in most cases, we represent them through a parametrized sigmoid function

$$\mathcal{W}_t = \mathcal{W}_0 + \frac{\delta_{\mathcal{W}}}{1 + \exp^{-\xi(t-\tau_0)}},$$

where $\mathcal{W}_0$ is the initial mixture coefficient, $\delta_{\mathcal{W}}$ is the difference between the initial and final mixture coefficients, ξ is the learning rate and τ_0 is the transition point. For further analysis, we characterize the $\widetilde{w}_t$ curves through the parameter vector $\Theta = (\mathcal{W}_0, \delta_{\mathcal{W}}, \xi, \tau_0)$.

Simulation-based validation

We formulated a response-tracking analysis to derive the participants' learning curves. We use these curves to identify prototypical profiles of fast and slower learners. To validate this approach, we designed a synthetic learner for which we controlled (i) the speed of learning and (ii) memory-order transition time. Our logic was to control the parameters of the synthetic learner, and then test whether we can recover information about these parameters using response-tracking modeling.

The synthetic learner was presented with sequences viewed by human participants, and was tasked with predicting the upcoming symbol. The learner purposefully had a different architecture from the Markov models used to generate the stimulus sequences. In particular, the learner extracted n-grams; i.e., blocks of consecutive symbols of length L = 1 (unigram), 2 (bigram) or 3 (trigram). During training, it learnt the n-gram probability distribution:

$$P_{t_s}(\mathcal{U}) = (1 - \gamma) \cdot P_{t_s - 1}(\mathcal{U}) + \gamma \cdot \delta(\mathcal{U}, \widehat{\mathcal{U}}),$$

where $\mathcal{U}$ denotes n-grams, t_s is the stimulus index, $\widehat{\mathcal{U}}$, is the observed n-gram at time t_s, $\gamma \in (0, 1)$ is a learning rate, and $\delta(\cdot, \cdot)$ is the delta function[1].

For all levels, the initial n-gram probabilities were uniformly distributed. At any given time the synthetic learner learnt a distribution of n-grams of a fixed length L, however the n-gram length changed over the course of training. For level-0 sequences, learning was initially driven by random guessing (all length-1 n-grams were equally probable); as training proceeded (after N_0 blocks of level-0 sequences) the synthetic learner started to learn n-gram distribution of length L = 1. For level-1 sequences, learning was initially driven by length L = 1 n-grams; as training proceeded (after N_1 blocks of level-1 sequences) the synthetic learner started to learn n-gram distribution of length L = 2. For level-2 sequences, learning was initially driven by length L = 2 n-grams; as training proceeded (after N_2 blocks of level-2 sequences) the synthetic learner started to learn n-gram distribution of length L = 3.

These n-gram distributions were used to generate predicted symbols as follows: If the model had no memory (L = 1; i.e. context length = 0), the synthetic learner drew a random sample from the n-gram distribution. If the context is first or second order ($L > 1$), after presenting $L - 1$ symbols (context $\mathcal{C}$) before the target, there were four possible n-grams $\mathcal{U}s$ of length L, depending on the target symbol prediction $s \in \{A, B, C, D\}$. The n-gram $\mathcal{C}$ containing the target prediction s was then chosen in a probabilistic manner:

$$\mathcal{C}s \sim \frac{P(\mathcal{C}s)}{\sum_{s' \in \{A,B,C,D\}} P(\mathcal{C}s')}, \; s \in \{A, B, C, D\}.$$

That is, the target was predicted to be symbol s with probability $P(s|\mathcal{C})$.

We applied response-tracking modeling to the responses of the synthetic learner. In particular, we fixed the memory-order transition times N_0, N_1 and N_2 to 1, 1 and 3 blocks, respectively, and varied the learning rate. We then applied the response-tracking modeling to the synthetic learner's responses and obtained the mixture coefficient curves $\widetilde{w}_t$ for the correct memory order. To investigate the relation between learning rate and the mixture coefficient curves $\widetilde{w}_t$, we computed the mean curve $\overline{w}_t(\gamma)$ averaged across all stimulus sequences of the same level (i.e. memory order). **Fig S3** shows that for all levels (level-0, level-1, and level-2), the slopes of the mean curves $\overline{w}_t(\gamma)$ increase monotonically with training. Moreover, similar to our participants, some synthetic learners extract the correct context length for a given level faster than others (given an appropriate change in the synthetic learner's learning rate). These simulations validate our response-tracking modeling, by demonstrating that using this modeling approach we were able to recover memory-order (i.e. context length) from synthetic data.

[1] $\delta(\mathcal{U}, \widehat{\mathcal{U}})$ = 1 if $\mathcal{U} = \widehat{\mathcal{U}}$ and $\delta(\mathcal{U}, \widehat{\mathcal{U}})$ = 0 otherwise

Predictive contingency

In addition to learning the context length, the participants need to learn the context-target contingencies. To test to how participants learn context-target contingencies, we used predictive contingency modeling (2). For each level, we compared: (i) the true underlying Markov model used to generate sequences (i.e. level-0, level-1 and level-2) and (ii) an alternative, less complex model (i.e. a random guessing model for level-0, an approximate marginalized level-0 model for level-1 and an approximate marginalized level-1 model for level-2).

To quantify the difference at trial t between a baseline model Q and the predictive contingency model P_t obtained by tracking the participants' responses with the mixture model (2), we used the expected Kullback-Leiber (KL) divergence from Q to P_t :

$$\mathcal{KL}_t = \sum_{\mathcal{C}} P(\mathcal{C}) \cdot \text{KL}\Big(Q(s|\mathcal{C}) || P_t(s|\mathcal{C})\Big).$$

The overall difference between Q and P_t is thus computed as a weighted average of the context-specific differences (KL-divergences) over all contexts in the baseline model. Here, $Q(s|\mathcal{C})$ is the context-conditional probability of the next symbol following context $\mathcal{C}$ in Q, $P_t(s|\mathcal{C})$ is the participant's predictive contingency for that context at trial t, and $P(\mathcal{C})$ is the theoretical distribution of contexts for Q obtained from the data generating model. The smaller the divergence between the predictive contingency model and a baseline model, the greater their similarity (in information theoretic terms). Recall that for level-0 stimulus sequences, empty context $\emptyset$ (of zero length) is the only context in the baseline model. This implies $P(\emptyset) = 1$ and $Q(s|\emptyset) = Q(s)$, which leads to

$$\mathcal{KL}_t = \text{KL}\Big(Q(s) || P_t(s|\emptyset)\Big).$$

We then computed the difference between the expected KL divergence from the less complex model to the predictive contingency model and the expected KL divergence from the true underlying Markov model to the predictive contingency model. We refer to this quantity as ΔKL, measuring which of the two baseline models is closer (in the information theoretic sense) to the participant's predictive contingency model. Positive values of ΔKL indicate that participant responses approximated the true Markov model used to generate sequences, while negative values indicate responses were based on a less complex model.

Extracting temporal structure using an entropy-based approach

Our response-tracking modeling approach is based on applying different order Markov models to the participants' responses. A potential concern with this approach is that it may over-constrain our understanding of the learning process. As an alternative less constrained approach, we adapted and applied a model for temporal sequence learning [1] based on acquiring a library of symbol sequences (i.e., 'engrams'). The original Jensen et al model acquired a library of engrams using a pruning process based on the entropy of the next-symbol distributions, conditional on the context provided by the preceding symbols. In particular, their algorithm monitored the stream of symbols and computed the entropy of engrams up to seven symbols long (i.e. context order 7). If an engram had low entropy (i.e., the last symbol was predicted almost perfectly by the preceding symbols) it was stored in the dictionary, while contexts with high entropies (i.e., unpredicted by the previous symbols) were pruned out. In this way, the

algorithm implemented by Jensen et al was able to use its acquired dictionary to predict upcoming symbols without formal understanding of the generative process that produced the sequence.

Directly applying this method to the sequences in our study, however, was unsuccessful. The Jensen et al model was designed and tested using sequences that were more deterministic than those used in our study. Having minimal stochasticity provides a ready means of pruning the acquired library of contingencies; in our case this fails because our probabilistic sequences were more random, meaning that a simple entropy rule did not provide a ready basis for pruning.

We therefore modified the entropy-based approach to fit the context of our study that uses probabilistic sequences and unsupervised learning. In doing this, we were limited in the context length we could consider. Our human participants learned while making relatively few responses (i.e., we have a sparse dataset), meaning that an attempt to extract context lengths of >2 would lead to insufficient model regularization. As the Jensen et al work did not test human learning, this problem was obviated: their simulations used 1000 item sequences repeated 100 times.

We considered unigrams and bigrams preceding the target as contexts. For all contexts $\mathcal{C}$ in the library, we tracked the next-symbol entropies H($\mathcal{C}$) across training blocks. Using these entropy time series, we derived an entropy-based measure for each context. For each immediate context (e.g. level-1 context A), there exists a number of higher-order contexts (i.e. level-2 contexts AA, BA, CA, and DA). To assess whether a level-1 context A is appropriate, we computed the following measure: ΔH(A) = H(A) - (H(AA) + H(BA) + H(CA) + H(DA)) / 4. If ΔH(A) is positive, the next-order symbol distribution of context A is less informative than the average information level given by its extended level-2 contexts AA, BA, CA, and DA. Hence higher-order contexts are more appropriate. If ΔH(A) is negative, context A is sufficiently informative and should be preferred to higher-order contexts.

Implementing this entropy-based model for level-2 data (**Fig S4**; Experiment 1, Group 0) indicates that the participants used the correct contexts for the prediction tasks (i.e. the first-order contexts A and D; second-order contexts AB, XB, BC, and YC). This reproduces the results produced by our response-tracking modeling using a less-constrained approach. However, as the entropy-based model requires a larger number of trials to extract learning strategies from participant responses, its use in tracking learning dynamics is limited. In particular, we could only apply the entropy-based method in the relatively saturated learning regime after the 10th block of participants' responses; that is, there were insufficient participant responses to reliably estimate entropy in the first 10 blocks.

Functional clustering analysis

Inspecting **Fig 3a** suggested prototypical differences in mixture coefficient curves $\widetilde{w}_t$ across participants. To identify prototypical patterns, we used k-means clustering. We first initialized the clustering algorithm by generating n cluster prototypes using randomly selected n learning curves and fitting a sigmoid function to each cluster. To partition the $\widetilde{w}_t$ curves, we defined the Euclidean distance D_{ij} between a $\widetilde{w}_t$-curve $\widetilde{w}_t^j$ and a sigmoid prototype $\mathcal{W}_t^i$:

$$D_{ij} = \frac{1}{T_j} \cdot \sum_{t=1}^{T_j} \left(\widetilde{w}_t^j - \mathcal{W}_t^i \right)^2,$$

where T_j is the length of $\widetilde{w}_t^j$, $\mathcal{W}_t^i$ stands for the value of sigmoid function specified by parameter Θ_i and evaluated at t. The cluster index for $\widetilde{w}_t^j$ is then $i_j^* = \arg\min_{i\in\{1,\ldots,n\}} D_{ij}$.

Using these initialized cluster prototypes, the clustering algorithm reassigned individual learning curves into one of the clusters, and then updated the prototypes by re-estimating their parameters Θ_i. The parameter Θ_i is obtained by minimizing $\sum_{j\in J_i} D_{ij}$, where $J_i = \{j|i_j^* = i\}$. Note that this assumes that the differences between $\widetilde{w}_t$-curves and the sigmoid prototype are Gaussian distributed with constant variance. This is equivalent to assuming that the noise model on the prototypes is homoeostatic and Gaussian. To monitor the clustering quality, the Sum of Squared Errors (SSE) is used to measure how well the $\widetilde{w}_t$-curves within a cluster are represented by the prototype

$$\text{SSE} = \sum_{i=1}^{n} \sum_{j=1}^{|J_i|} T_j \cdot D_{ij}.$$

We searched for prototypical sigmoidal functions that give rise to the smallest average SSE. As the clustering process is stochastic, we repeated initialization ten times and selected the solution with minimal SSE. We also repeated the process of prototype re-estimation fifty times to ensure convergence. To determine the number of clusters needed to capture the data, we evaluated how SSE decreases with increasing n. Specifically, the rate of SSE reduction should drop sharply when the number of clusters exceeds the optimal number of clusters n_{opt}. Therefore, n_{opt} can be identified as the 'knee' position in the n-SSE curve [2]. We contrasted the SSE against the number of clusters and assessed the statistical significance using a Bayesian Information Criterion (BIC) [3] that penalised large number of clusters:

$$\text{BIC}(n) = \text{SSE} + 4n \cdot \log\left(\sum_{i=1}^{n} \sum_{j=1}^{|J_i|} T_j\right).$$

Note that $4n$ is the number of free parameters in n sigmoid prototypes, while $\sum_{i=1}^{n} \sum_{j=1}^{|J_i|} T_j$ is the number of observations in the data. Such regularisation favors a smaller number of clusters, subject to sufficiently small SSE, giving $n_{\text{opt}} = \arg\min_n \text{BIC}(n)$. Using this approach, we found a knee at n = 2 in the SSE curves for level-0, level-1, and level-2 (**Fig S2**), indicating that two clusters best described the data.

References

[1] Jensen, S., Boley, D., Gini, M., Schrater, P. 2005 Rapid on-line temporal sequence prediction by an adaptive agent. In *Proceedings of the fourth international joint conference on Autonomous agents and multiagent systems* (pp 67–73), ACM.

[2] Foss, A., Zaane, O. R. 2002 A parameterless method for efficiently discovering clusters of arbitrary shape in large datasets. In *Proceedings of the 2002 IEEE International Conference on Data Mining* (pp 179–186), IEEE Computer Society.

[3] Greg, H., Charles, E. 2004 Learning the k in k-means. *Advance in neural information processing systems* **17**, 117-149.

[4] Kass, R. E., Raftery, A. E. 1995 Bayes factors. *Journal of American Statistical Association* **90**, 773–795 (DOI:10.2307/2291091).

Figure S1

a

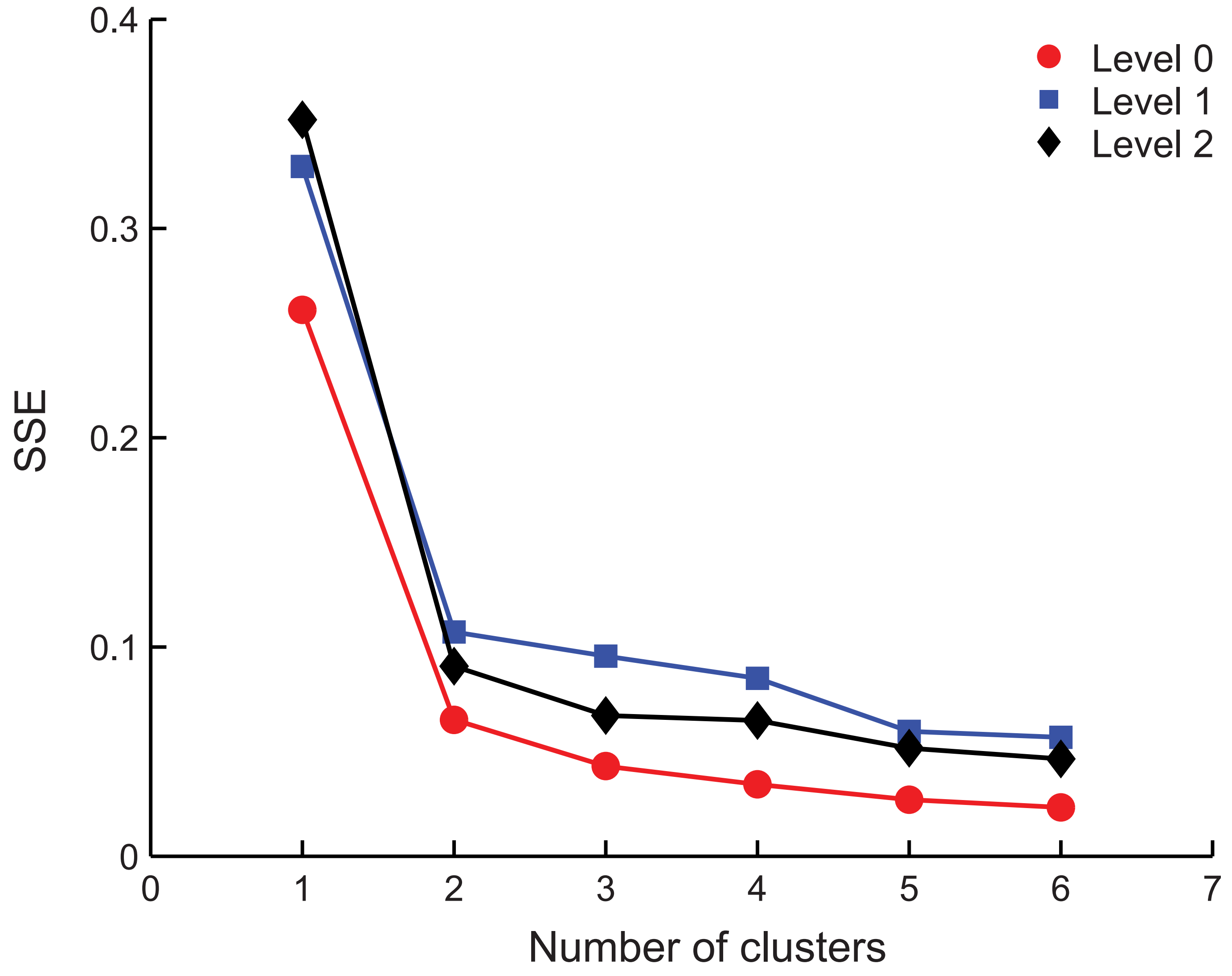

b

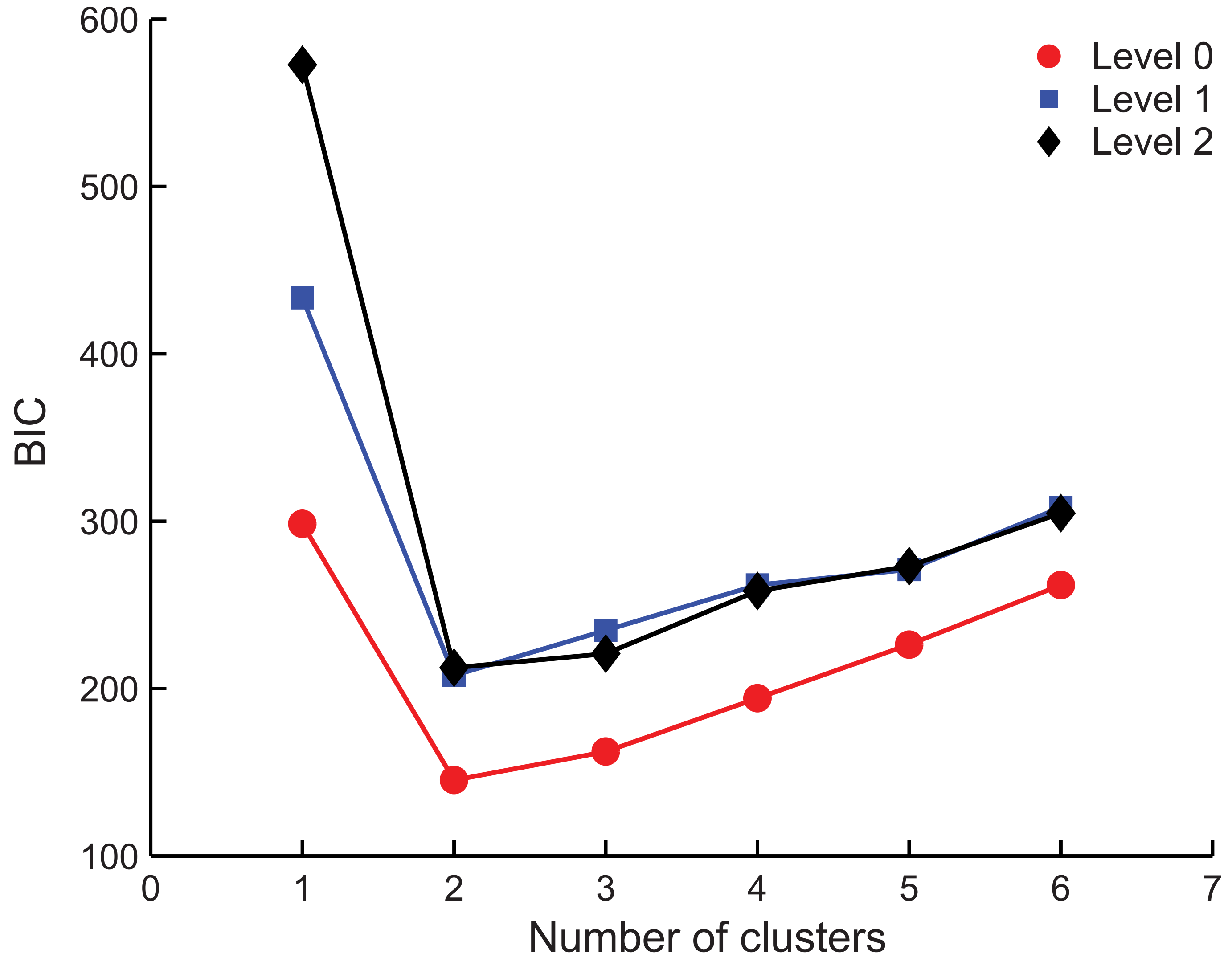

Figure S1: Experiment 1: functional clustering analysis. Data are shown for level-0, level-1 and level-2. (a) Plot of SSE (Sum of Squares Error) against number of clusters n. The optimal number of clusters is identified as the 'knee' position in the *n*-SSE curve. (b) Plot of BIC (Bayesian Information Criterion) against number of clusters *n*. The optimal number of clusters is identified as the minimum position in the *n*-BIC curve. In particular, we calculated the overall SSE for different number of clusters *n* by computing the 'optimal' *n* (n_{opt}), and plotting the curve of SSE against *n*. A knee at nopt indicates that clustering accuracy (as measured by SSE) initially increases quickly (with *n* less than n_{opt}), while it becomes insignificant when the cluster number exceeds nopt. Results from this analysis were confirmed using an additional model selection method, Bayesian Information Criterion (BIC) to determine the optima number of clusters. Statistical significance of n_{opt} clusters is quantified by the difference between BIC($n = n_{opt}+1$) and BIC($n = n_{opt}$), denoted as ΔBIC. ΔBIC > 6 indicates statistical significance [4]. Thus, $n_{opt} = 2$ was statistically significant across levels: level-0: ΔBIC=17.1; level-1: ΔBIC=26.9; level-2: ΔBIC=8.5.

Figure S2

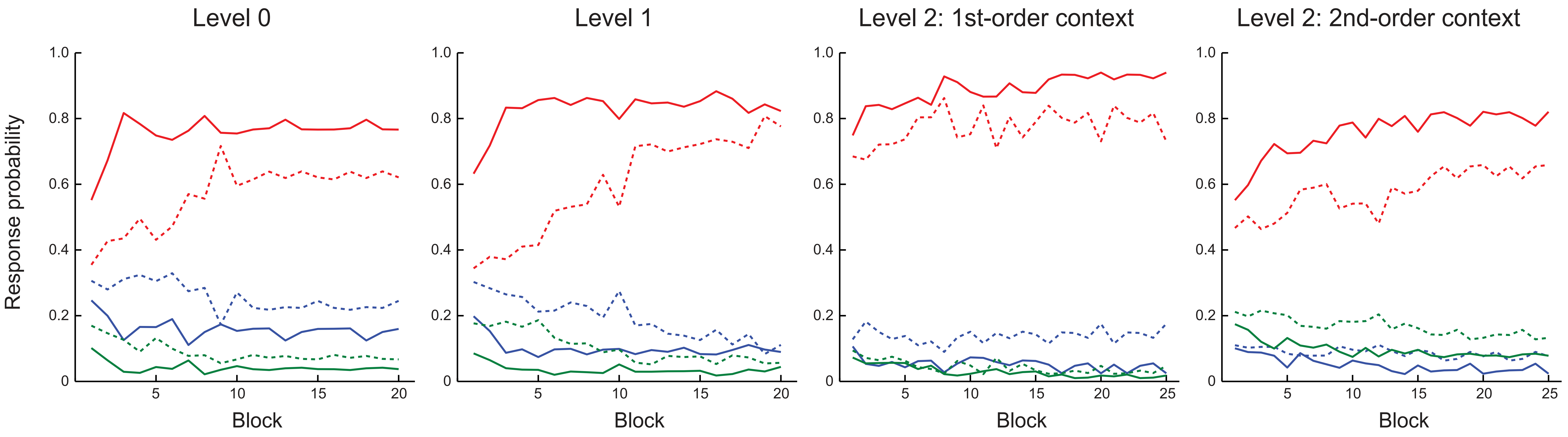

Figure S2: Experiment 1 Response probabilities. Response probabilities for individual targets (level-0) or conditional probabilities of context-target contingencies (level-1, level-2) across training blocks. Red lines indicate targets or context-target contingencies with the highest (conditional) probability (0.72 for level-0 and 0.8 for level-1 or level-2), blue lines indicate the second highest (conditional) probabilities (0.18 for level-0 and 0.2 for level-1 or level-2), and green lines indicate targets or context-target contingencies that appear rarely (0.05). For level-2, 1st- (Context A/D) and 2nd-order contexts (Context XB/YC/AB/BC) are presented separately. Solid lines: fast learners; dashed lines: slower learners.

Figure S3

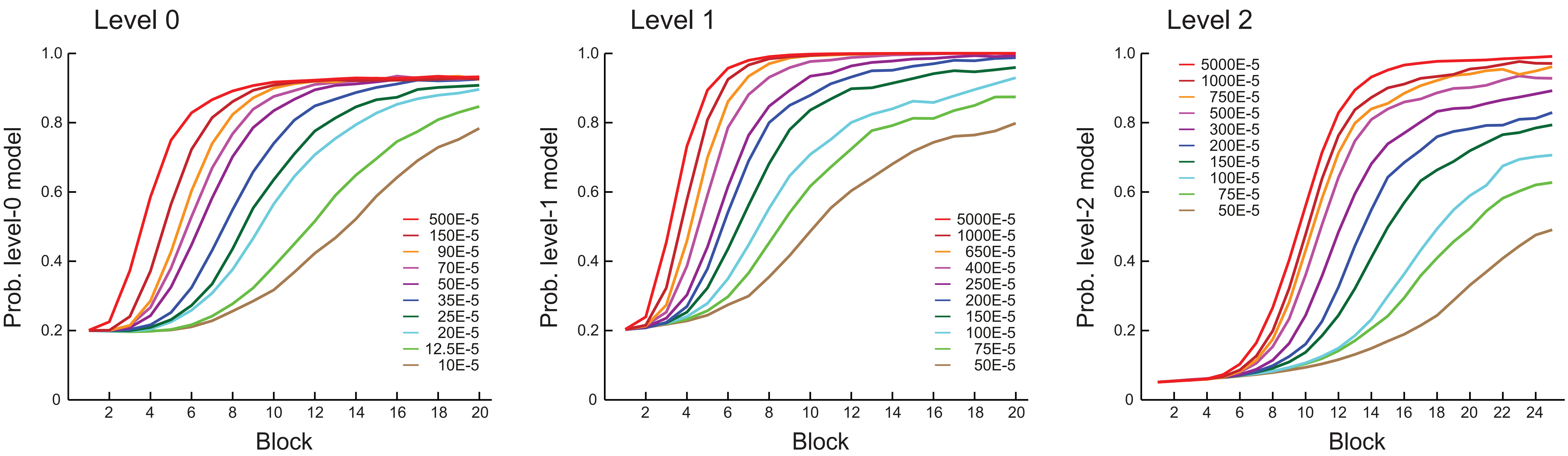

Figure S3: simulation-based validation. Learning curves (i.e. mixture coefficient curves) for synthetic learners trained on level-0, level-1 and level-2 sequences. The memory-order transition times N_0, N_1 and N_2 were fixed to 1, 1, and 3 blocks, respectively. A range of learning rate values were tested and a control parameter   was used to specify how quickly the synthetic learners extracted the correct context length per level.

Figure S4

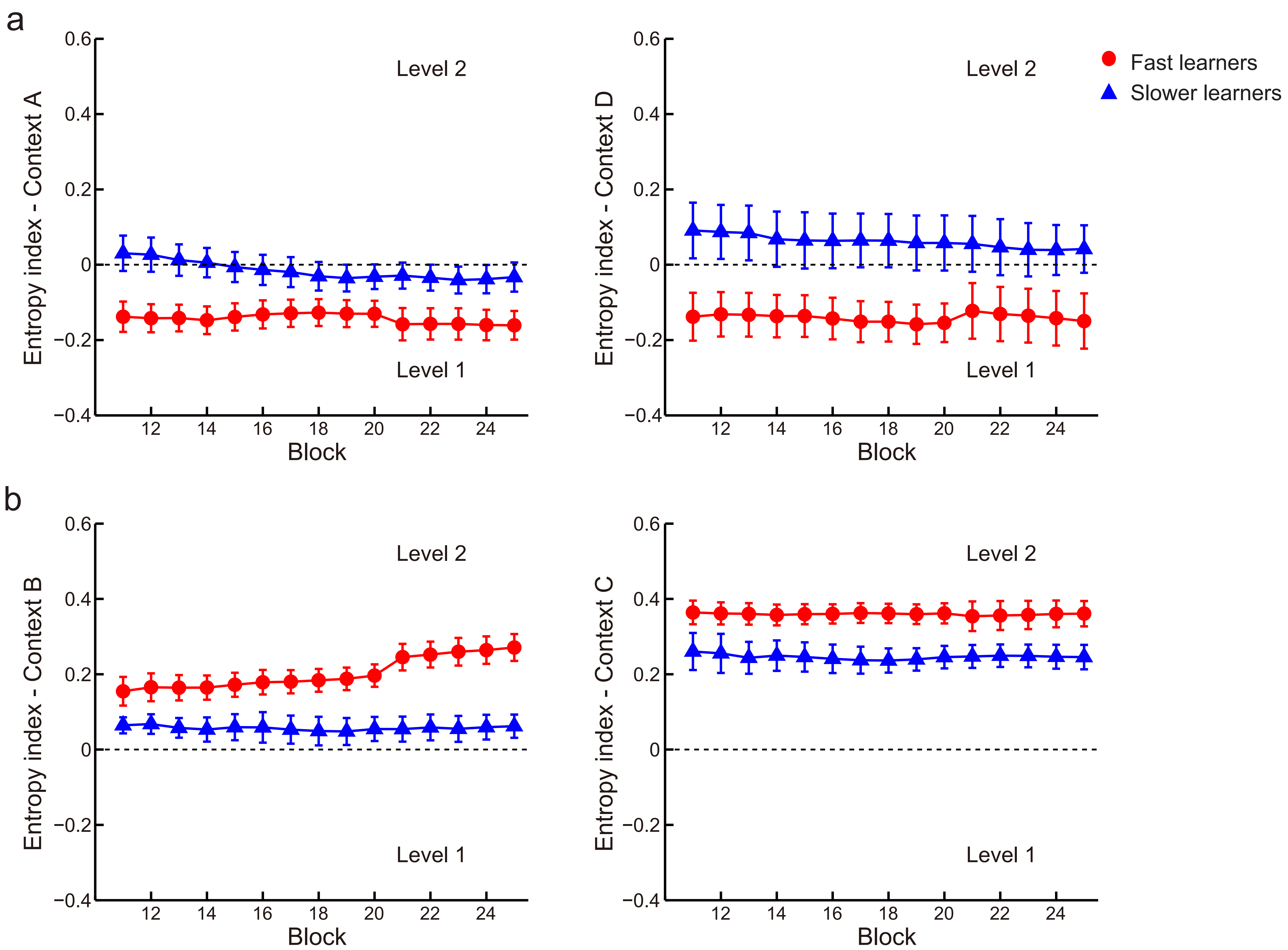

Figure S4: entropy based modeling (A) Entropy index (ΔH) across training blocks for context A (left panel) and D (right panel). Participant responses were analysed with an entropy-based model for level-2 sequences (Experiment 1, Group 0). Entropy index values below zero indicate that the first-order context has higher predictive power (i.e. explains better the participants' responses) than second-order contexts (e.g. AA, BA, CA, and DA for context A; AD, BD, CD, and DD for context D). Data from first 10 blocks could not be used because there were insufficient participant responses to reliably estimate entropy. Average data is shown across participants (i.e. fast learners cluster in red vs. slower learners cluster in blue). (B) Entropy index (ΔH) across training blocks for context B (left panel) and C (right panel); these are second-order contexts for level-2 sequences.

Figure S5

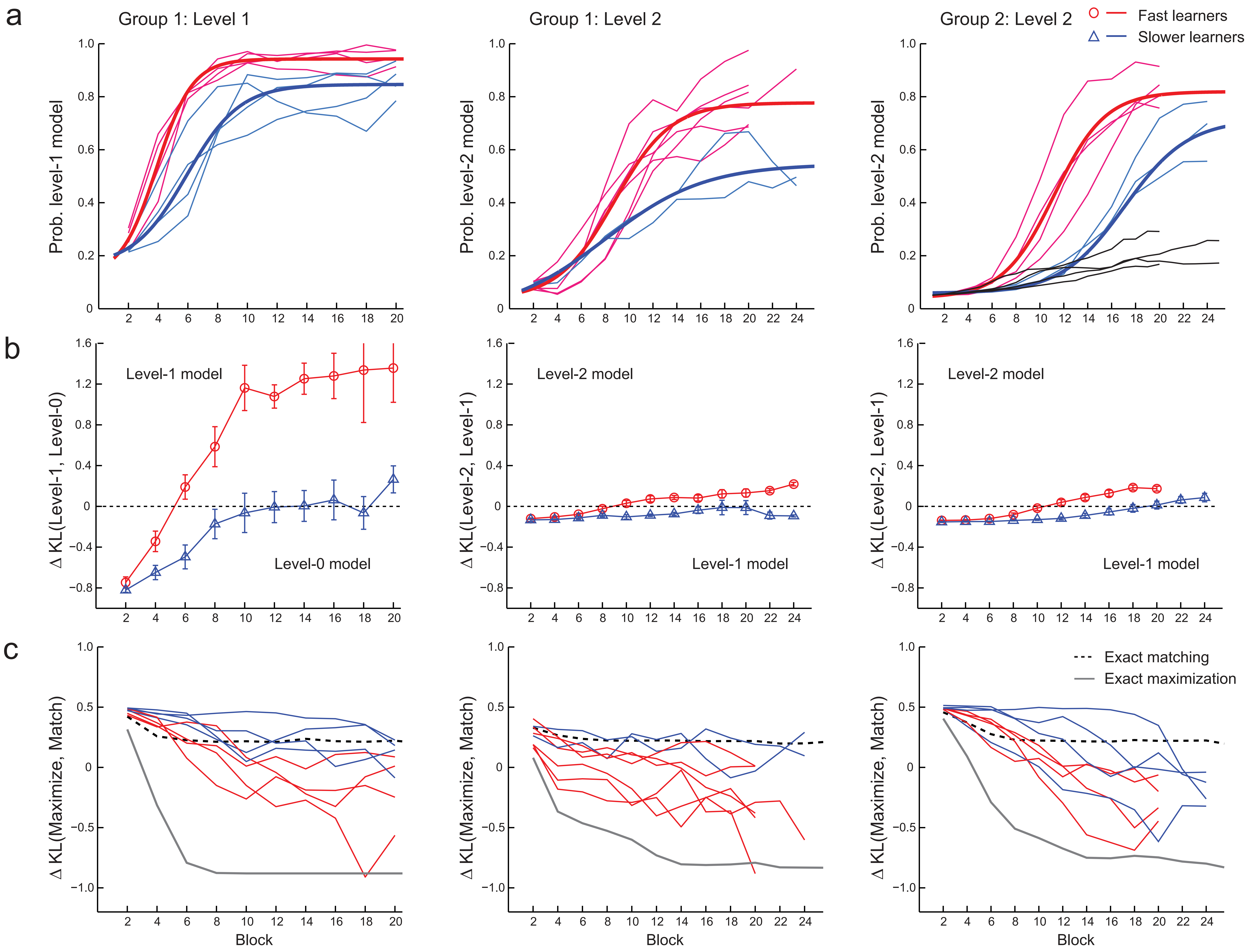

Figure S5: Experiment 2: modeling. Data is shown for Group 1 (n = 8): level-1 and level-2; and Group 2 (n = 8) separately for each participant cluster (i.e. red vs. blue). (a) Mixture coefficient curves for each individual participant. Two prototypical clusters are identified using functional clustering analysis: red (Group 1: level-1: n = 4, level-2: n = 6; Group 2: n = 4) vs. blue (Group 1: level-1: n = 4, level-2: n = 2; Group 2: n = 4). Data is also shown for four participants in Group 2 ('others') who showed less than 25% probability of extracting the correct model order at the end of training and therefore they were not considered in further analysis. (b) ΔKL-curves based on the comparison of response-based models (predictive contingency model) and baseline models per level averaged for each participant cluster. Error bars indicate SEM. (c) Strategy choice as indicated by ΔKL comparing two possible strategies (matching vs. maximization) is shown for each individual participant. Exact matching and maximization models are indicated by black dashed lines and grey solid lines, respectively.

Figure S6

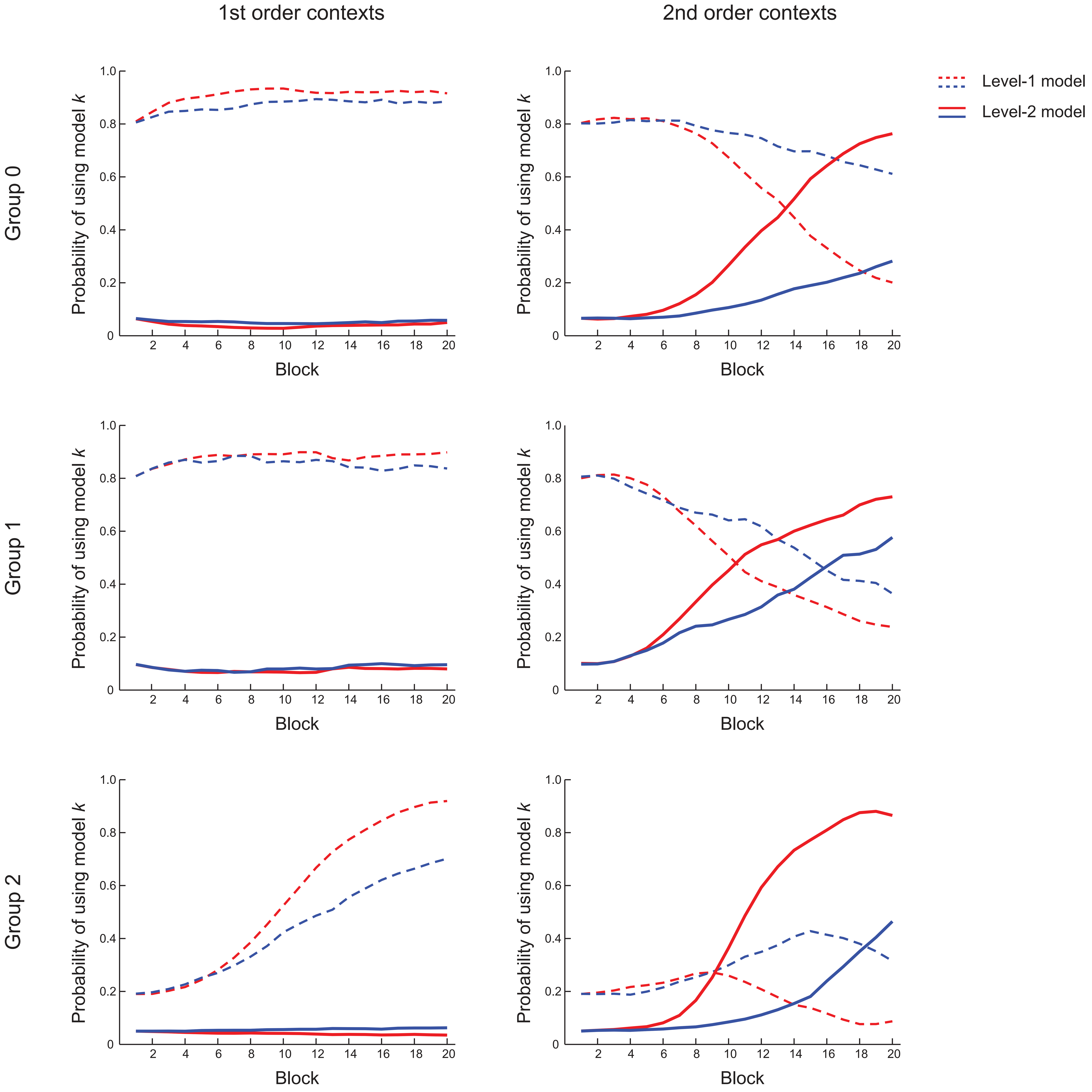

Figure S6: learning 1st- vs. 2nd- order context for level-2 sequences. Probability of using level-1 (dashed lines) and level-2 (solid lines) models for level-2 sequences that are determined by 1st- vs. 2nd-order contexts. Mean data are shown for fast learners (red) vs. slower learners (blue) in Group 0, Group 1 and Group 2, for 1st- and 2nd- order contexts. For level-2, we separated 1st- from 2nd- order trials based on the context order preceding the target in each trial. We then derived a context length model for each set of trials and computed the probability of using level-1 vs. level-2 models for each trial. This analysis showed that for 1st- order trials, there was no difference between individuals with different learning profiles (as characterized by functional clustering analyses) in Groups 0 and 1. In contrast, the responses of Group 2 participants could be explained by a 1st-order model; that is, participants used 1st-order context to predict stimuli that were preceded 2nd-order contexts during the initial phase of learning. Interestingly, fast learners started using 2nd-order contexts earlier than slower learners who continued to rely on 1st-order contexts. Further, this analysis showed that learning 1st-order contexts intermixed with 2nd-order contexts (as in the variable context length model used for level-2) is more difficult than learning 1st-order contexts presented alone (as in Group 1, level-1). That is, participants extracted the correct 1st-order context length earlier in the training when they were exposed only to level-1 sequences (Group 1, level-1, Fig S5a) than when they were presented with variable context length sequences (Group 2, level-2, Fig S6). This suggests that learning variable context length sequences is more difficult not only because of the complexity of higher-order structures but also because participants have to extract more than one context length.

Figure S7

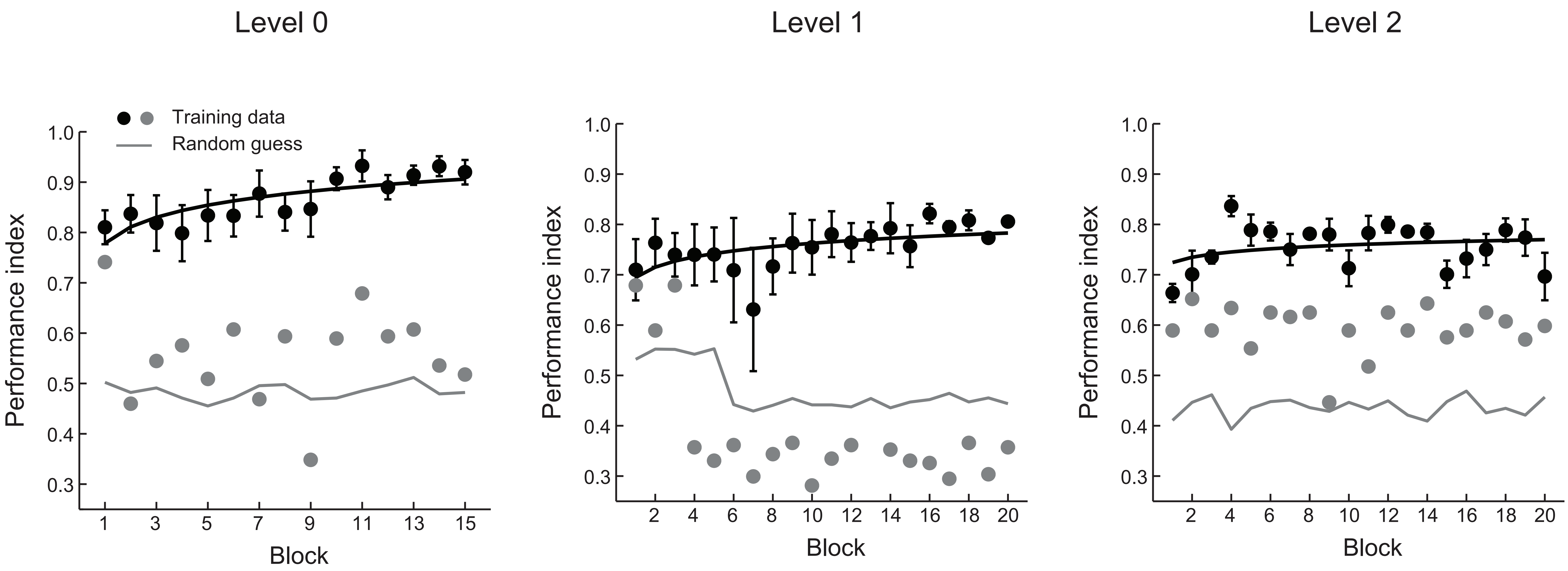

Figure S7: No-feedback control experiment. Performance index is shown across training blocks and levels. Fitted data is shown for participants who improved during training (black circles, level-0, n = 8; level-1, n = 6; level-2, n = 3). There were a small number of participants who did not improve during training (grey circles, level-0, n = 1; level-1, n = 1; level-2, n = 1). The random guessing baseline is indicated by dashed lines.
